# Intestinal neuropod cell GUCY2C regulates visceral pain

**DOI:** 10.1172/JCI165578

**Published:** 2023-02-15

**Authors:** Joshua R. Barton, Annie K. Londregan, Tyler D. Alexander, Ariana A. Entezari, Shely Bar-Ad, Lan Cheng, Angelo C. Lepore, Adam E. Snook, Manuel Covarrubias, Scott A. Waldman

**Affiliations:** 1 Department of Pharmacology, Physiology, & Cancer Biology,; 2 Department of Neurosciences,; 3 Department of Microbiology & Immunology, and; 4 Sidney Kimmel Cancer Center, Thomas Jefferson University, Philadelphia, Pennsylvania, USA.

**Keywords:** Gastroenterology, Neuroscience, Guanylate cyclase, Neuroendocrine regulation, Pain

## Abstract

Visceral pain (VP) is a global problem with complex etiologies and limited therapeutic options. Guanylyl cyclase C (GUCY2C), an intestinal receptor producing cyclic GMP(cGMP), which regulates luminal fluid secretion, has emerged as a therapeutic target for VP. Indeed, FDA-approved GUCY2C agonists ameliorate VP in patients with chronic constipation syndromes, although analgesic mechanisms remain obscure. Here, we revealed that intestinal GUCY2C was selectively enriched in neuropod cells, a type of enteroendocrine cell that synapses with submucosal neurons in mice and humans. GUCY2C^hi^ neuropod cells associated with cocultured dorsal root ganglia neurons and induced hyperexcitability, reducing the rheobase and increasing the resulting number of evoked action potentials. Conversely, the GUCY2C agonist linaclotide eliminated neuronal hyperexcitability produced by GUCY2C-sufficient — but not GUCY2C-deficient — neuropod cells, an effect independent of bulk epithelial cells or extracellular cGMP. Genetic elimination of intestinal GUCY2C amplified nociceptive signaling in VP that was comparable with chemically induced VP but refractory to linaclotide. Importantly, eliminating GUCY2C selectively in neuropod cells also increased nociceptive signaling and VP that was refractory to linaclotide. In the context of loss of GUCY2C hormones in patients with VP, these observations suggest a specific role for neuropod GUCY2C signaling in the pathophysiology and treatment of these pain syndromes.

## Graphical abstract

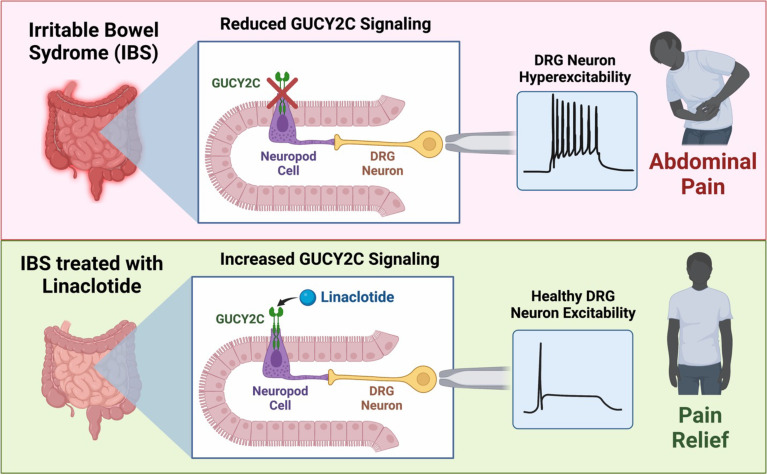

## Introduction

Visceral pain (VP) is a debilitating and prevalent condition, affecting 25% of adults, resulting in over 20 million outpatient visits annually (1, 2). The most common causes of VP are irritable bowel syndrome (IBS) and inflammatory bowel disease (IBD), which have complex etiologies and a paucity of therapeutic options (3, 4). While opioids are often prescribed to relieve pain, they can exacerbate disease, decrease quality of life, and lead to tolerance and dependence (5). There is an unmet need for new nonopioid analgesics to provide safe and effective treatments for patients with VP (5, 6).

Guanylyl cyclase C (GUCY2C), a brush-border transmembrane-receptor enzyme expressed by epithelial cells throughout the intestine, has emerged as a novel therapeutic target in VP (7). Endogenous GUCY2C agonists uroguanylin (GUCA2B) in the small intestine and guanylin (GUCA2A) in the colorectum bind to the extracellular domain of GUCY2C and induce the intracellular catalytic domain to produce cyclic GMP (cGMP) (8–10). This cyclic nucleotide regulates protein kinase G II (PRKG2), which drives intestinal fluid secretion (11, 12). In that context, the synthetic GUCY2C agonists linaclotide and plecanatide are approved for oral treatment of chronic constipation syndromes like constipation-type IBS (IBS-C) and chronic idiopathic constipation (CIC) (13–15).

Beyond constipation, clinical studies revealed that oral GUCY2C agonists linaclotide and plecanatide relieve VP in patients with IBS-C (16–22). Similarly, oral or intra-rectal GUCY2C agonists reduce VP elicited by colorectal balloon distension (CRD) in rodents after, but not before, chemically induced sensitization by colorectal inflammation (18–21, 23). Analgesia produced by those agonists required GUCY2C expression, and they were without effect on VP in GUCY2C-deficient mice (19–23). GUCY2C-agonist relief of VP was associated with reduced firing of colonic visceral–afferent nerves and decreased activation of ascending-nociceptive signaling in the spinal dorsal horn quantified by neuronal phosphorylation of ERK (pERK) (19–23).

The mechanism for GUCY2C agonist–induced analgesia has remained elusive. Current hypotheses posit that GUCY2C agonists induce bulk intestinal epithelial cells to secrete extracellular cGMP, which serves as a neuromodulator of submucosal visceral afferent nerves, inhibiting pain signaling (19–23). Indeed, extracellular application of cGMP reduced the firing of colonic visceral afferent nerves in ex vivo preparations and increased the rheobase, or threshold for the neurons to fire (22). However, this model is incomplete, and the mediator of extracellular cGMP’s inhibitory action on the visceral afferent nerve fibers has not yet been identified (22).

Here, we identify a rare (< 1%) type of epithelial cell selectively enriched in the proximal small intestine of mice and humans that over expresses GUCY2C. These GUCY2C^hi^ cells differentially express markers of EECs and neurons, but not GUCY2C hormones or the machinery characterizing fluid and electrolyte secretion. Further, they extend basal pseudopodia into the lamina propria in close apposition to submucosal neurons. Indeed, morphologic, transcriptomic, and protein analyses suggest that GUCY2C^hi^ cells are recently identified neuropod cells that mediate afferent and efferent synaptic transmission between the gut and central nervous system (24–28). Neuropod cells are a subtype of enteroendocrine cell first identified by their unique morphology, including basal pseudopods, and their interaction with neurons and glia (25, 26). Subsequent studies revealed that neuropod cells form functional synapses with peripheral neurons in coculture and transmit signals directly to neurons through canonical neurotransmitters (24, 27, 28). Similarly, GUCY2C^hi^ intestinal cells establish functional connections with cocultured dorsal root ganglion (DRG) neurons. Under basal conditions without GUCY2C agonists, GUCY2C^hi^ cells spontaneously amplify DRG-neuron excitability. Conversely, the GUCY2C agonist linaclotide eliminates this excitability in DRG neurons only when DRG neurons are cocultured and interacting with GUCY2C^hi^ cells. The inhibitory effect of linaclotide is not recapitulated in cocultures with GUCY2C-deficient neuropod cells or by administering extracellular cGMP. Moreover, selectively eliminating GUCY2C expression in neuropod cells in mice produced a spontaneous VP syndrome and abolished the ability of oral linaclotide to relieve VP.

Together, these observations suggest that GUCY2C in GUCY2C^hi^ small intestinal neuropod cells dynamically modulates afferent-neuron excitability. Moreover, they suggest that these cells specifically mediate the analgesia produced by oral GUCY2C agonists, through a mechanism that dampens neuron excitability produced by neuropod cells. Because GUCY2C hormones are lost in chronic-constipation syndromes (29), it is tempting to speculate that the resultant attenuation of GUCY2C paracrine signaling in neuropod cells could contribute to the pathophysiology of VP in those conditions.

## Results

### GUCY2C was overexpressed by rare, morphologically distinct, intestinal cells.

**Figure 1:**
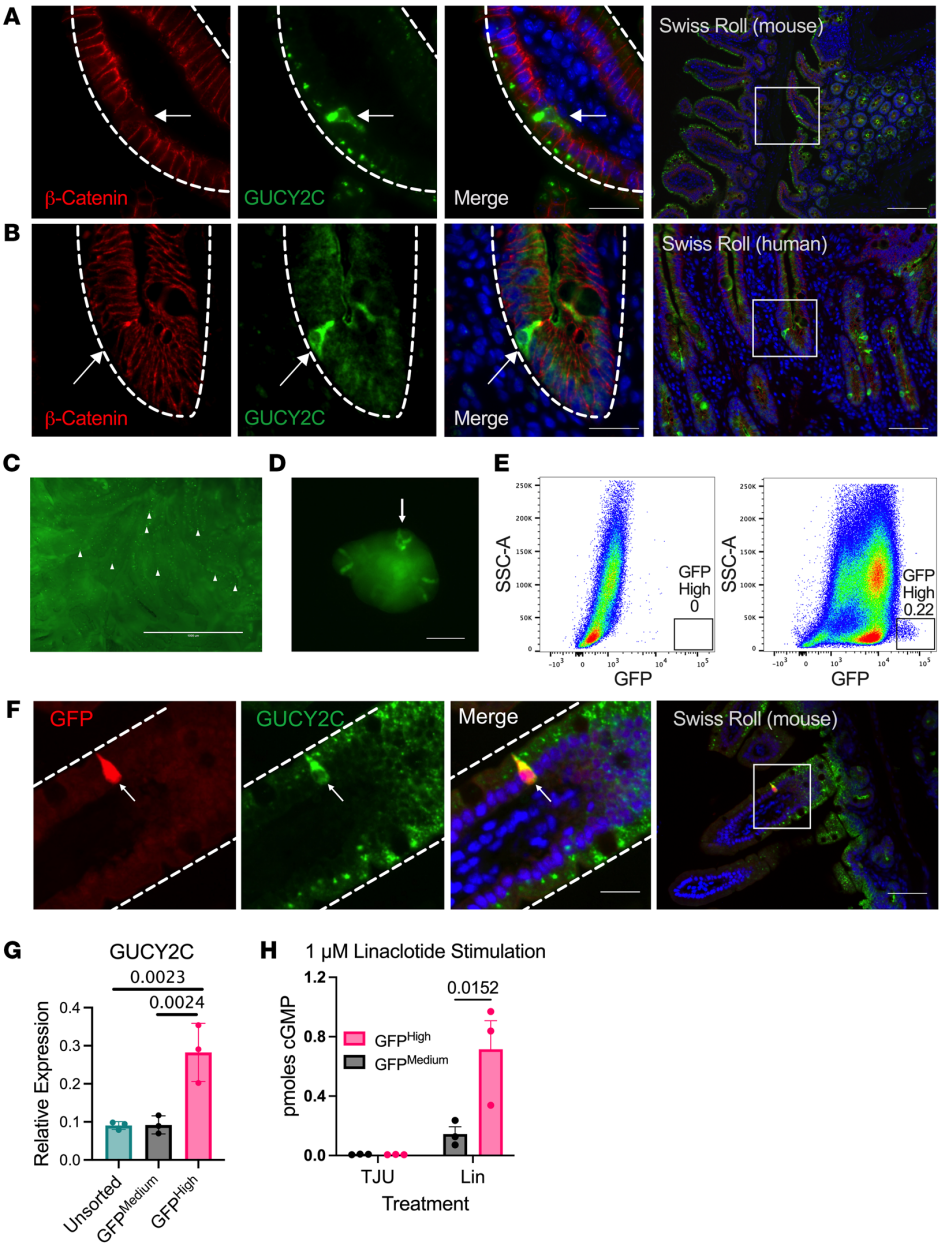
GUCY2C is enriched in a subset of intestinal cells. (**A** and **B**) Immunofluorescence of mouse jejunum (**A**) or human duodenum (**B**) reveals intestinal cells with increased GUCY2C expression and basal pseudopods (arrows). White dashed lines delineate intestinal epithelium. (**C** and **D**) Live images of GUCY2C-GFP reporter mouse show GFP expression throughout the intestine, punctuated with brightly fluorescent cells with basal pseudopods (arrows) in freshly dissected intestine (**C**) and intestinal organoids (**D**). (**E**) FACS of dissociated GUCY2C-GFP mouse intestinal epithelium (right) compared with nonfluorescent intestine (left) reveals a small population of cells with increased GFP fluorescence intensity (GFP High) (representative of n = 3). (**F**) GUCY2C-GFP^hi^ cells in the GUCY2C-GFP reporter mouse have increased GUCY2C expression, as shown by immunofluorescence. (**G**) GFP^hi^ cells are enriched in GUCY2C transcripts compared with bulk intestinal epithelial cells (20,000 cells/sample, 2 samples/mouse, n = 3 mice). (**H**) The GUCY2C agonist linaclotide induces cGMP production by sorted GFP^hi^ cells (10,000 cells/sample, 2 samples /mouse, n = 3*)*. Scale bars: 25 μm (Merge), 100 μm (Swiss Roll). Statistics calculated using 2-way ANOVA with Tukey’s multiple comparisons test.

GUCY2C is heterogeneously produced by the intestinal epithelium, with overexpression (GUCY2C^hi^) seen in rare mucosal cells in mice and humans (Figure 1, A and B). GUCY2C^hi^ cells have a distinct morphology compared with other intestinal epithelial cells, with extension of basal pseudopods into the submucosa (Figure 1, A and B). Individual cells overexpressing GUCY2C were visualized as distinct puncta in the intestinal mucosa of novel transgenic mice expressing GFP driven by the GUCY2C promoter (GUCY2C-GFP; Figure 1C). Cells with distinct pseudopodia overexpressing GFP developed in enteroids prepared from GUCY2C-GFP mice (Figure 1D). GUCY2C and GFP overexpression precisely coincided in the mucosa of GUCY2C-GFP mice, and GFP^hi^ cells purified by FACS were rare [0.22% + 0.5 (n = 3)] (Figure 1, E and F). FACS-sorted GUCY2C-GFP^hi^ cells overexpressed GUCY2C mRNA (Figure 1G) and produced exaggerated cGMP responses to the GUCY2C agonist linaclotide (Figure 1H) compared with bulk intestinal epithelial cells (GFP^med^). Flow cytometry of intestinal epithelium revealed that these cells are concentrated in the small intestine compared with the colorectum and are distributed throughout the crypt-villus axis (Supplemental Figures 1 and 2; supplemental material available online with this article; https://doi.org/10.1172/JCI165578DS1). These observations reveal what we believe to be a previously unknown rare population of intestinal epithelial cells with a unique morphology and amplified GUCY2C expression and function.

### GUCY2C^hi^ cells differentially expressed enteroendocrine and neuronal transcripts.

**Figure 2:**
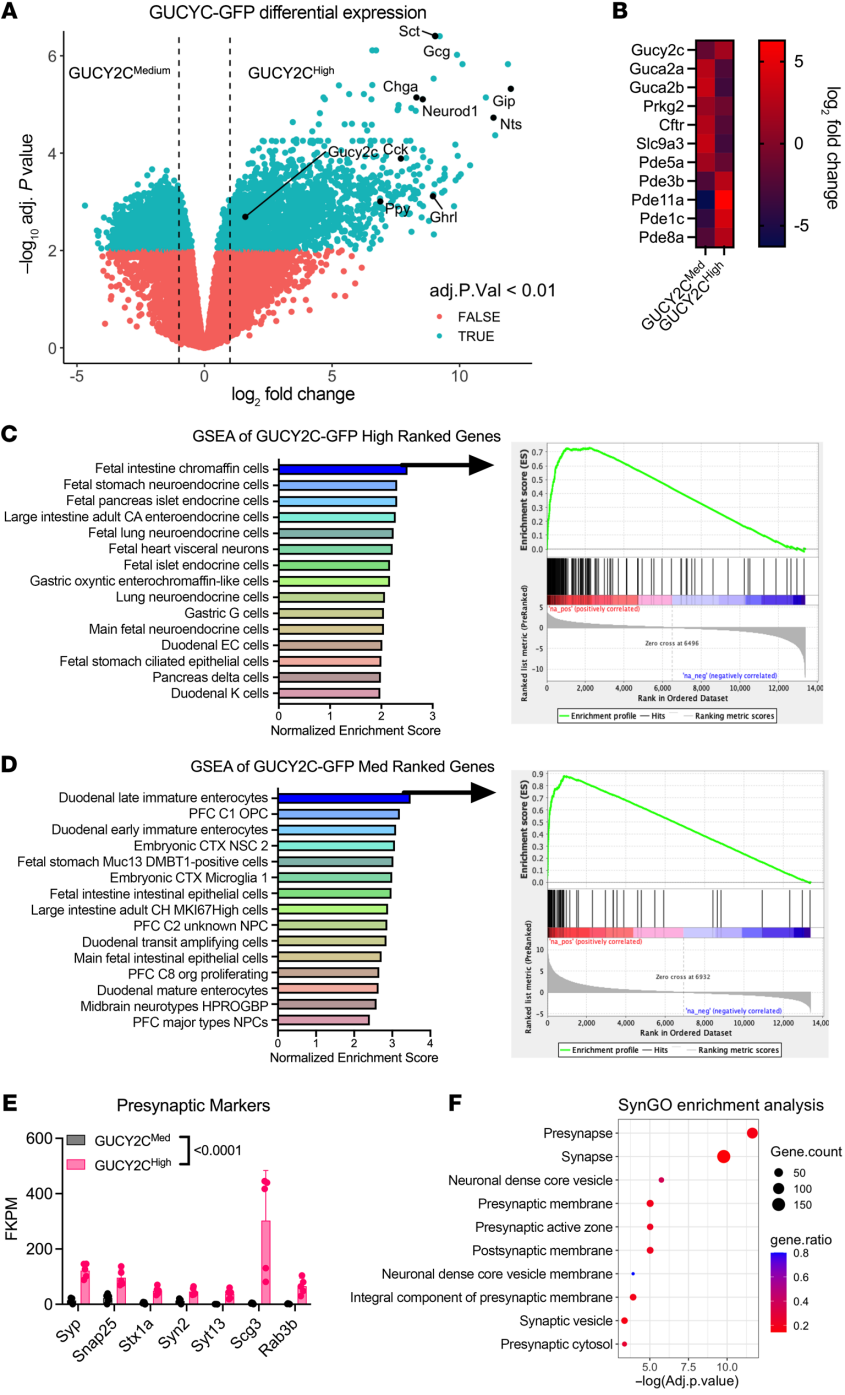
GUCY2C^hi^ cells differentially express enteroendocrine and neuronal transcripts. (**A**) RNA sequencing of sorted GUCY2C^hi^ and GUCY2C^med^ cells from GUCY2C-GFP small intestine reveals that the transcripts most enriched in GUCY2C^hi^ cells are markers of enteroendocrine cells. (20,000 cells/group; n = 5) (**B**) GUCY2C^hi^ cells are enriched in *Gucy2c*, but not in GUCY2C ligands *(Guca2a, Guca2b*) or canonical downstream targets of GUCY2C secretory signaling *(Prkg2, Cftr, Slc9a3,* and *Pde5a*). (**C**) GSEA reveals that GUCY2C^hi^ cells exhibit a neuroendocrine phenotype, while (**D**) GUCY2C^med^ cells phenocopy enterocytes (15 gene sets with highest normalized enrichment scores shown, all FDR<0.0001) (**E**) GUCY2C-GFP^hi^ cells are enriched in the presynaptic marker transcripts enriched in ChrgA^+^ ECs. (**F**) SynGO analysis reveals that GUCY2C-GFP^hi^ cells are enriched in transcripts of presynaptic and postsynaptic proteins (GO terms with padj < 0.05). Statistics for **A** and **B** were determined through Limma (v3.40.6) and edgeR (v3.26.8). GSEA analysis was performed on gene sets with differential expression adjusted *P* values < 0.01. Statistics for **E** were calculated using 2-way ANOVA with Tukey’s multiple comparisons test.

Transcriptomic profiling revealed that the most differentially upregulated genes in GUCY2C-GFP^hi^ cells characterize intestinal enteroendocrine cells (EECs), especially their hormone products, which include Pyy, Nts, Gcg, Sct, Gip, Chga, Cck, and Ghrl (Figure 2A) (30). While GUCY2C-GFP^hi^ cells are enriched in GUCY2C, they are deficient in canonical GUCY2C paracrine ligands and the downstream targets of cGMP signaling mediating fluid and electrolyte secretion, compared with bulk GUCY2C-GFP^med^ or GUCY2C^med^ epithelial cells (Figure 2B). Gene Set Enrichment Analysis (GSEA) demonstrated that GUCY2C^hi^ cells produced transcripts associated with neuroendocrine cells (Figure 2C), compared with GUCY2C^med^ cells, which express transcripts associated with enterocytes (Figure 2D). Surprisingly, GUCY2C^hi^ cells also were enriched in transcripts that are characteristic of neuronal cells, especially those contributing to the formation of synapses and electrical excitability (Figure 2E and Supplemental Figure 3) (24). Indeed, Synaptic Gene Ontology (SynGO) analysis of upregulated genes in GUCY2C^hi^ cells (*P* adjusted > 0.01) revealed upregulation of pre and postsynaptic gene products (Figure 2F, Supplemental Figure 4, and Supplemental Data File) (31). These observations suggested that GUCY2C^hi^ cells share properties of both EECs and neurons. Their unique profile aligns with recently identified neuropod cells that link the gut and central nervous system through both endocrine hormones and synaptic neurotransmission (25, 26, 32).

### GUCY2C was overexpressed by neuropod cells.

**Figure 3:**
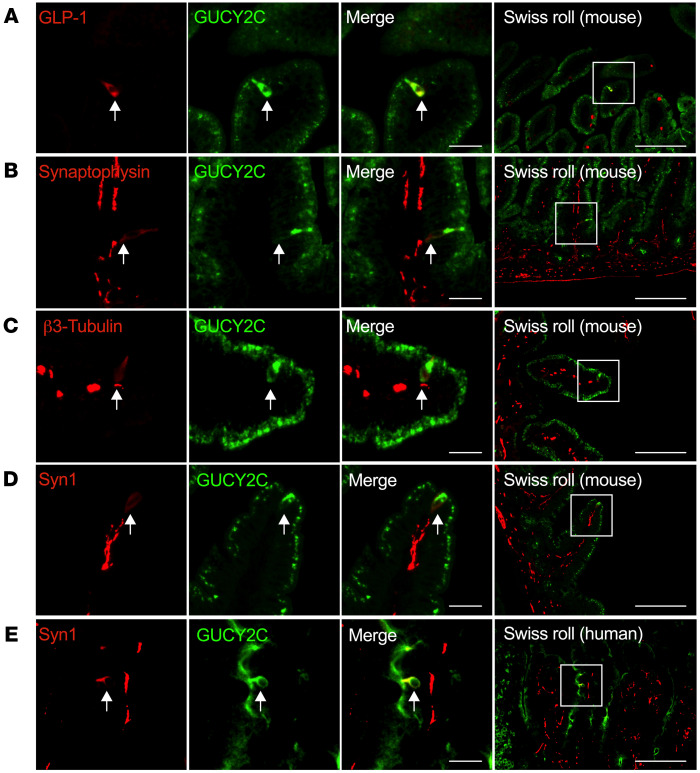
GUCY2C^hi^ cells coexpress markers of EECs and neuropod cells and interact with submucosal neurons. GUCY2C high cells express markers of (**A** and **B**) EECs (GLP-1 and synaptophysin) and (**C** and **D**) neuronal markers (B3 tubulin and Syn1) in mouse duodenum (arrows). (**E**) Human GUCY2C-GFP^hi^ cells also are enriched for Syn1, a neuropod marker in duodenum (arrows). Scale bars: 25 μm (Merge), 100 μm (Swiss Roll).

Neuropod cells are specialized EECs that express endocrine and neuronal gene products and have a unique morphology, projecting basal neuropods into the submucosa (25, 26, 32) that synapse with vagal and DRG neurons (24, 28). Indeed, GUCY2C^hi^ cells expressed the EEC products glucagon-like peptide1 (Glp-1) and synaptophysin (Figure 3, A and B). The original studies characterizing neuropod cells used the presynaptic protein synapsin1 (Syn1) as a marker of intestinal epithelial cells with neuron-like properties (24–28). Accordingly, GUCY2C^hi^ cells express the neuropod marker Syn1, as well as β3-Tubulin, a cytoskeletal protein that characterizes neurons, as well as these neuron-like cells (Figure 3, C and E). GUCY2C^hi^ cells extend pseudopods into the lamina propria in close apposition to neurons, which also are characterized by their expression of synaptophysin, β3-tubulin, and Syn1 (Figure 3, B–D). Similarly, GUCY2C^hi^ cells in the human small intestine coexpress Syn1 and extended pseudopods into the lamina propria in apposition to Syn1-expressing neurons (Figure 3E).

**Figure 4:**
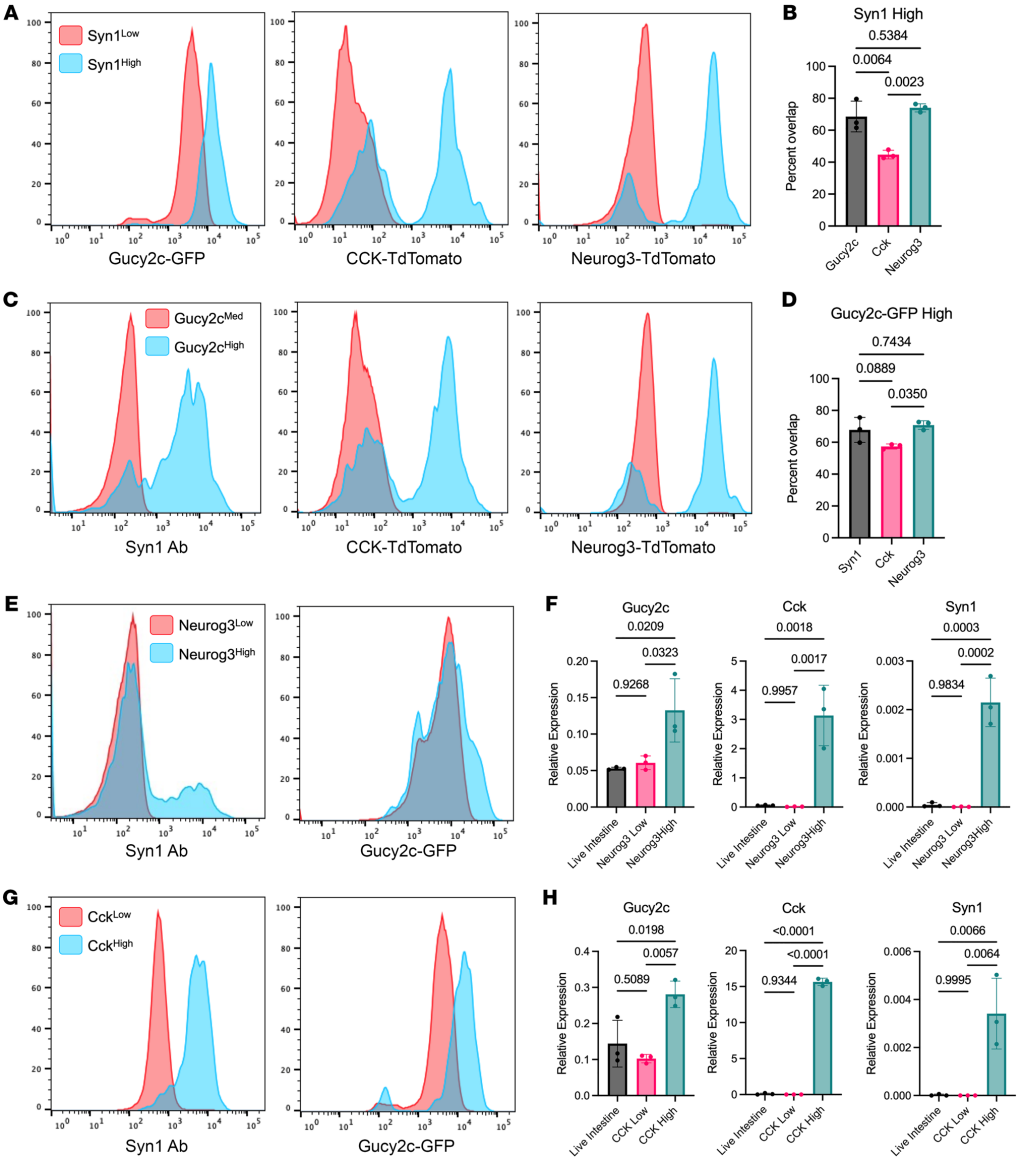
Neuropod cells and EECs are GUCY2C^hi^. All figures represent dissociated small intestinal epithelium stained with antibody, and are representative of at least 3 mice. (**A**) Syn1^hi^ cells (stained with Syn1 antibody) are enriched in GUCY2C, CCK, and emerged from a Neuorg3+ lineage in fluorescent mouse models compared with Syn1^lo^ cells. (**B**) Quantification of (**A**) reveals that a majority of Syn1^hi^ neuropod cells are GUCY2C^hi^, CCK^hi^, and Neurog3^hi^ (n = 3). (**C**) GUCY2C^hi^ cells (from GUCY2C-GFP mice) are enriched in Syn1 and CCK, and emerge from a Neuorog3+ lineage. (**D**) Quantification of (**C**), reveals that a majority of GUCY2C^hi^ cells are neuropod cells (Syn1^hi^), CCK^hi^, and Neuorg3^hi^ (n = 3). (**E**) A population of Neurog3^hi^ cells (from a *Neurog3-Tdtomato* mouse) are Syn1^hi^ and GUCY2C^hi^. (**F**) Neurog3^hi^ cells are enriched in Gucy2c, Cck (enteroendocrine marker), and Syn1 transcripts. (**G**) CCK^hi^ cells (from a *Cck-TdTomato* mouse) also are enriched Syn1^hi^ and GUCY2C^hi^. (**H**) Similarly, CCK^hi^ cells are enriched in Gucy2c, Cck (positive control), and Syn1 transcripts (n = 3). Statistics for **B**, **D**, **F**, and **H** were calculated using 1-way ANOVA with Tukey’s multiple comparisons test.

To evaluate the relative proportion of GUCY2C^hi^ cells that have neuropod-cell and enteroendocrine-cell properties, GUCY2C-GFP mice were crossed with *CCK^cre^-Tdtomato^fl/+^* mice, a fluorescent mouse model of neuropod cells, and *Neurog3^cre^-Tdtomato^fl/+^* mice (a fluorescent mouse model of enteroendocrine cells). Using Syn1 as a neuropod marker (24, 27, 28), 68.6% ± 9.6% of Syn1^hi^ cells overexpressed GUCY2C-GFP (Figure 4, A and B). This contrasts with the current mouse model of neuropod cells, *CCK^cre^-Tdtomato^fl/+^,* which only overlap with 44.7% **±** 2.7% of Syn1^hi^ cells (Figure 4, A and B) (28, 33). *Neurog3^cre^-Tdtomato^fl/+^-GUCY2C-GFP* mice (Supplemental Figure 5) leverage Neurog3 as a transcription factor required for EEC differentiation (34, 35). In these mice, 74.0% ± 2.6% of Syn1^+^ cells are Neurog3^hi^, implying that most neuropod cells differentiate along the EEC lineage (Figure 4, A and B). GUCY2C^hi^ cells substantially coincide with this profile, and 70.8% ± 2.7% of GUCY2C^hi^ cells were Neurog3^hi^, implying that they arose from EEC cell precursors (Figure 4, C and D). This agreed with transcriptomic analyses of Neurog3-expressing cells (36) and Glp-1–expressing cells (37) in which GUCY2C was overexpressed in mature EECs, as well as single-cell transcriptomic analyses demonstrating overexpression of GUCY2C by EECs in humans (Supplemental Figure 6) (38). Moreover, 67.8% ± 7.9% of GUCY2C^hi^ cells were Syn1^hi^, while 57.4% ± 1.6% coexpressed *Cck^cre^-Tdtomato^fl/+^* (Figure 4, C and D), leveraging Cck and Syn1 as markers for neuropod cells (27, 28). Sorting *Neurog3^cre^-Tdtomato^fl/+^* and *Cck^cre^-Tdtomato^fl/+^* mouse intestine revealed that GUCY2C transcripts were overexpressed in Neurog3^hi^ and Cck^hi^ cells (Figure 4, E–H). These data revealed that neuropod cells were a minority of enteroendocrine cells, and that some neuropod (Syn1^hi^) cells may have arisen from a population of cells that did not express Neurog3. However, because Neurog3 was expressed early in EEC development, some cells may express Neurog3 before expressing neuropod markers. Indeed, fate-mapping of Neurog3^+^ cells in Neurog3Chrono mice revealed that *Gucy2c* was upregulated only in mature EECs — cells that had expressed Neurog3 for more than 24 hours (36). Because our *Neurog3^cre^-Tdtomato* mice capture early, differentiating, and mature EECs, this phenomenon could partially explain why only a small proportion of Neurog3^+^ cells were GUCY2C^hi^ and Syn1^hi^. Together, these observations suggest that there is a specialized subpopulation of EECs (Neurog3^hi^) comprising neuropod (Syn1^hi^) cells with concurrent CCK (CCK^hi^) expression and GUCY2C (GUCY2C^hi^) overexpression (11.0% ± 3.9% of Neurog3^+^ cells are both Syn1^hi^ and GUCY2C^hi^, n = 3) that synapse with peripheral neurons (Supplemental Figure 5) (24, 28).

### GUCY2C modulated DRG-neuron excitability through neuropod cells.

**Figure 5:**
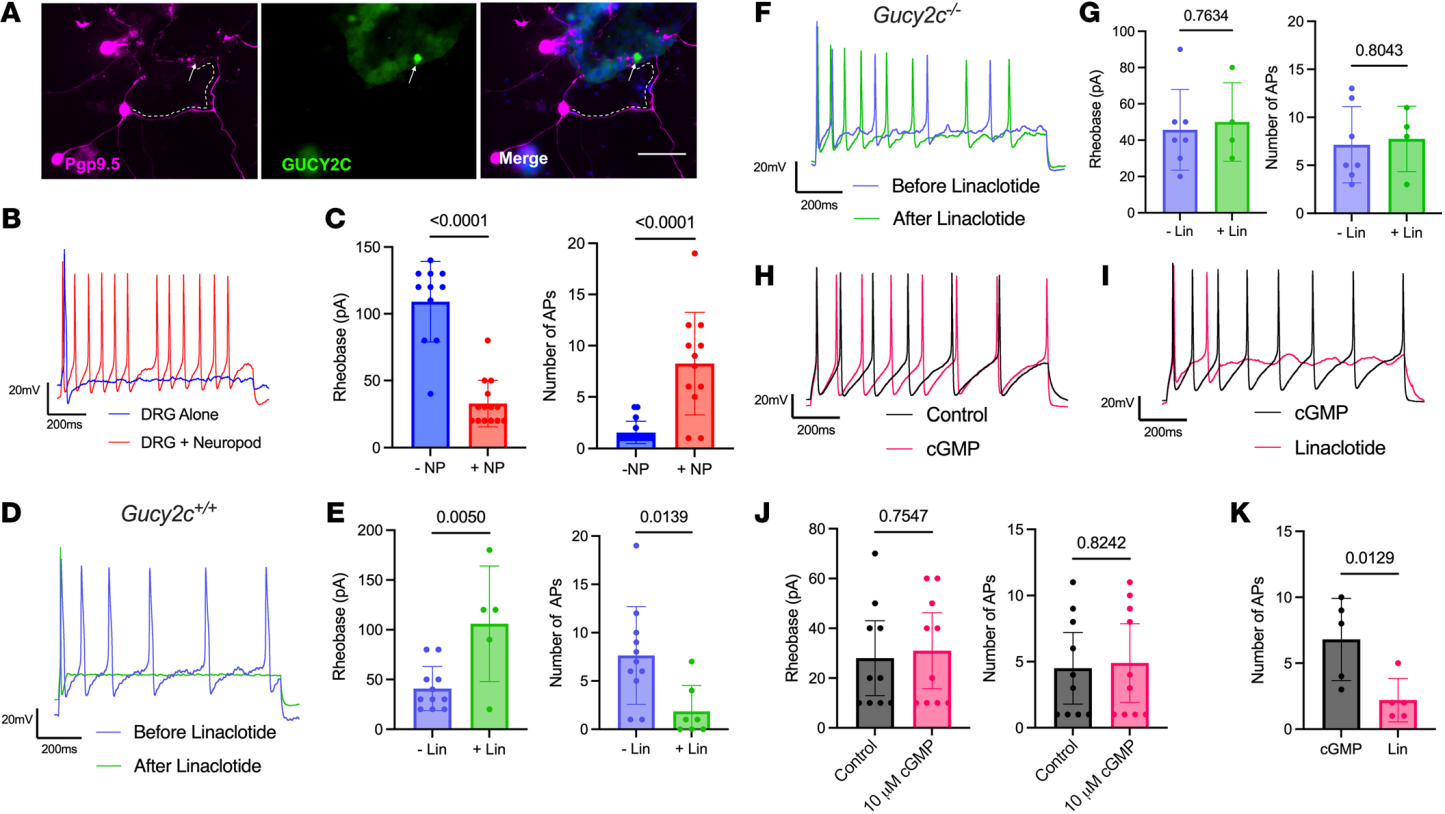
Neuropod cells regulate DRG neuron excitability through GUCY2C. (**A**) Immunofluorescence of E15.5 DRG neurons (Pgp9.5+) plated with small intestinal crypts from GUCY2C-GFP mice exhibit outgrowth directed specifically toward GUCY2C^hi^ neuropod cells 24 hours after plating. Scale bar: 50 μm. (**B**) Representative AP traces of stimulated DRG neurons in coculture with intestinal crypts. DRG neurons exhibit (blue) 1 AP when not near a neuropod (DRG Alone) or (red) repetitive firing when near a neuropod. (**C**) Summary of DRG neuron rheobase and number of APs fired under conditions **B** above. (**D**) Representative AP traces of DRG neurons + Neuropod before (blue) and after addition of 1 μM linaclotide (green), revealing loss of repetitive AP firing after linaclotide treatment. (**E**) Summary of DRG neuron rheobase and number of APs fired under conditions **E** above. (**F**) Representative AP traces of DRG neurons + Neuropod cells from *Gucy2c^–/–^* crypts before (blue) and after 1 μM linaclotide (green), reveal no change in repetitive firing. (**G**) Summary of DRG neuron rheobase and number of APs fired under conditions **F** above. (**H**) Representative AP traces of DRG cells + Neuropod cells showing repetitive AP firing before (black) and after 10 μM cGMP treatment (pink). (**I**) Representative AP traces of DRG neurons showing repetitive AP firing after 10 μM extracellular cGMP and decreased AP firing after subsequent 1 μM linaclotide. (**J**) Summary of DRG neuron rheobase and number of APs fired under conditions from **H** above. (**K**) Summary of DRG neuron rheobase and number of APs fired under conditions from **I** above. Representative traces represent 1 recording of the DRG neurons plotted in the subsequent rheobase and AP graphs. Statistics for **C**, **E**, **G**, **J**, and **K** were calculated using 2-tailed unpaired *t* test with an *f* test for comparisons of variances.

Neuropod cells in culture establish synapses with central and peripheral neurons that mediate neurotransmission (27, 28). Here, 14.4% of embryonic (E15.5) DRG neurons (68/473 DRG neurons counted over 17 coverslips) associated with GFP^hi^ or CCK^hi^ (GUCY2C^hi^) neuropod cells in coculture (Figure 5A and Supplemental Figure 7). DRG neurons in their basal state typically fired 1 evoked action potential (AP) at the onset of a sustained current–injection pulse with an intensity that corresponds to 3× rheobase. However, DRG neurons interacting with GUCY2C^hi^ neuropod cells without GUCY2C agonists were hyperexcitable, as demonstrated by the presence of repetitive spiking and a reduced rheobase (Figure 5, B and C). Conversely, activating GUCY2C with 1 μM linaclotide inhibited neuropod cell–induced DRG neuron hyperexcitability by returning the rheobase and AP firing to basal levels (Figure 5, D and E). Linaclotide had no effect on DRG neurons interacting with crypts isolated from *Gucy2c^–/–^* mice (Figure 5, F and G) indicating that effects on DRG-neuron excitability were mediated specifically by intestinal epithelial GUCY2C (Supplemental Figure 8). These observations suggest that neuropod cells modulated DRG neuron excitability, driving hyperexcitability when GUCY2C signaling was low but returning DRG neuron excitability to baseline when GUCY2C signaling was high. Mechanisms by which neuropod cell GUCY2C signaling modulated DRG neuron excitability remain to be defined. However, it was not by cGMP released from neuropod cells (19, 22) since linaclotide, but not extracellular cGMP, suppressed neuropod cell–induced DRG-neuron hyperexcitation (Figure 5, H–K).

### Silencing GUCY2C produced a spontaneous VP syndrome.

**Figure 6:**
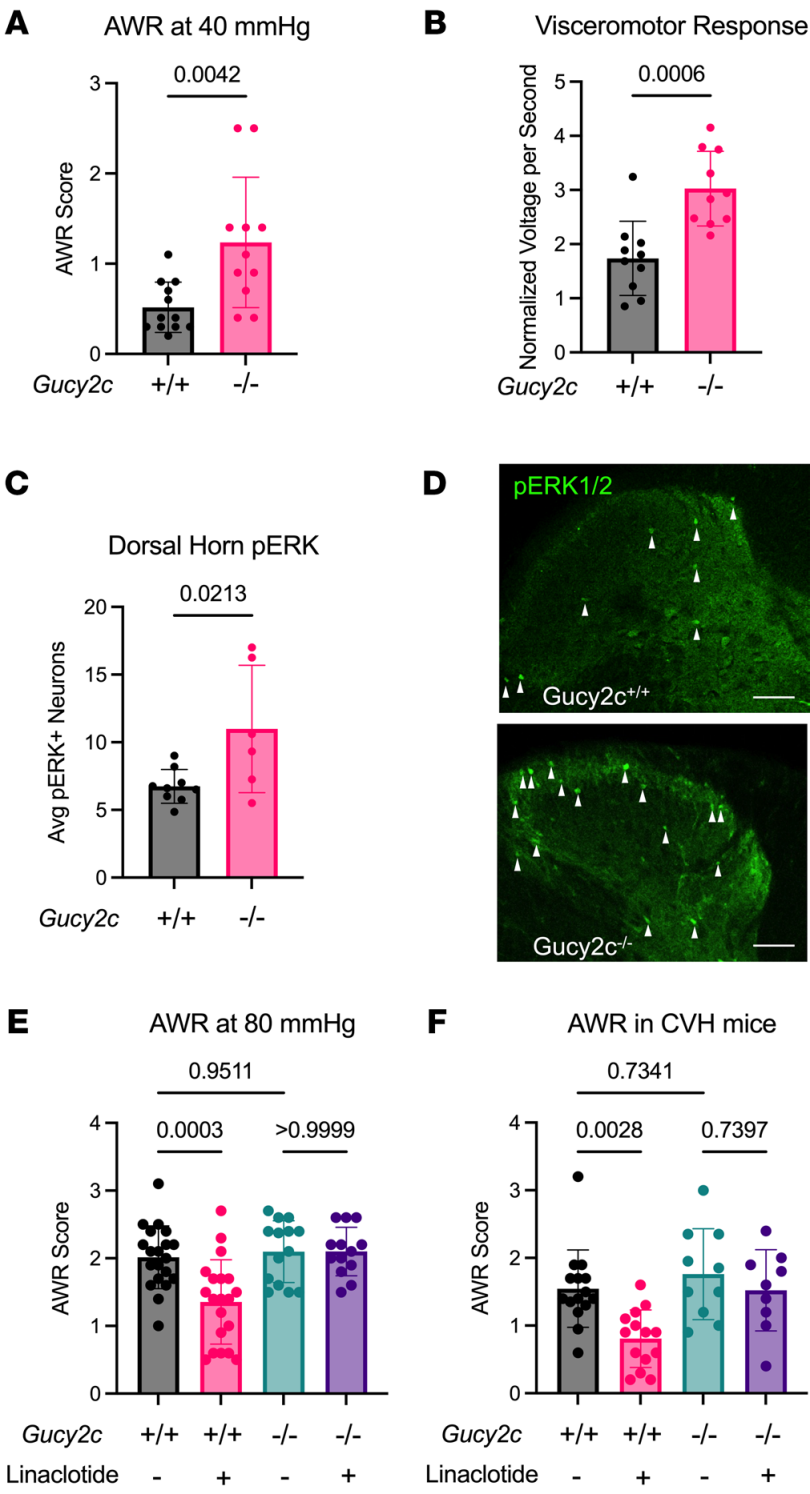
Gucy2c^–/–^mice exhibit a spontaneous VP syndrome that is refractory to linaclotide. (**A**) AWR, a behavioral readout of VP after CRD, reveals that *Gucy2c^–/–^* mice have a hyperalgesic response to nonnoxious CRD (40 mmHg; n = 11–12). (**B**) Visceromotor response of the external oblique reveals that *Gucy2c^–/–^* mice have increased pain reflexes compared with *Gucy2c^+/+^* mice (80 mmHg; n = 10). (**C**) Quantification of pERK^+^ cells in the dorsal horn of the thoracolumbar spinal cord reveals hyperalgesia in *Gucy2c^–/–^* mice after noxious CRD (80 mmHg; n = 6–9). (**D**) Representative images of pERK staining (arrowheads) in the dorsal horn of *Gucy2c^+/+^* and *Gucy2c^–/–^* mice. (**E**) AWR score of *Gucy2c^–/–^* and *Gucy2c^+/+^* mice after noxious CRD (80 mmHg) with and without 5 days of 4 μg/kg/day linaclotide gavage (n = 13–20). (**F**) AWR score of *Gucy2c^–/–^* and *Gucy2c^–+/+^* mice after nonnoxious CRD (40 mmHg) with and without 5 days of 4 μg/kg/day linaclotide gavage (n = 10–15). Statistics for **A**–**C** were calculated using 2-tailed unpaired *t* test with an *f* test for comparison of variances. Statistics for **E** and **F** were calculated using 2-way ANOVA with Tukey’s multiple comparisons test (all comparisons were included in statistical calculation, with only relevant comparisons shown for clarity).

GUCY2C hormone loss in patients with VP (29) suggests that GUCY2C signaling could play a role in the pathophysiology of VP. *Gucy2c^–/–^* mice were compared with *Gucy2c^+/+^* mice using behavioral (through abdominal withdrawal reflex; AWR), physiologic (through visceromotor reflex; VMR), and biochemical (through pERK) staining in the spinal cord readouts of VP. Silencing GUCY2C signaling genetically in *Gucy2c^–/–^* mice produced a spontaneous VP syndrome, amplifying AWR responses, VMR responses, and spinal cord pERK that mimicked colorectal exposure to 2,4,6-trinitrobenzenesulfonic acid (TNBS) (Figure 6, A–D). Conversely, oral linaclotide produced visceral analgesia in WT, but not *Gucy2c^–/–^*, mice (Figure 6E). Similarly, linaclotide relieved VP in *Gucy2c^+/+^*, but not *Gucy2c^–/–^,* mice exposed to TNBS (Figure 6F and Supplemental Figure 9).

### GUCY2C in neuropod cells selectively controlled VP.

**Figure 7:**
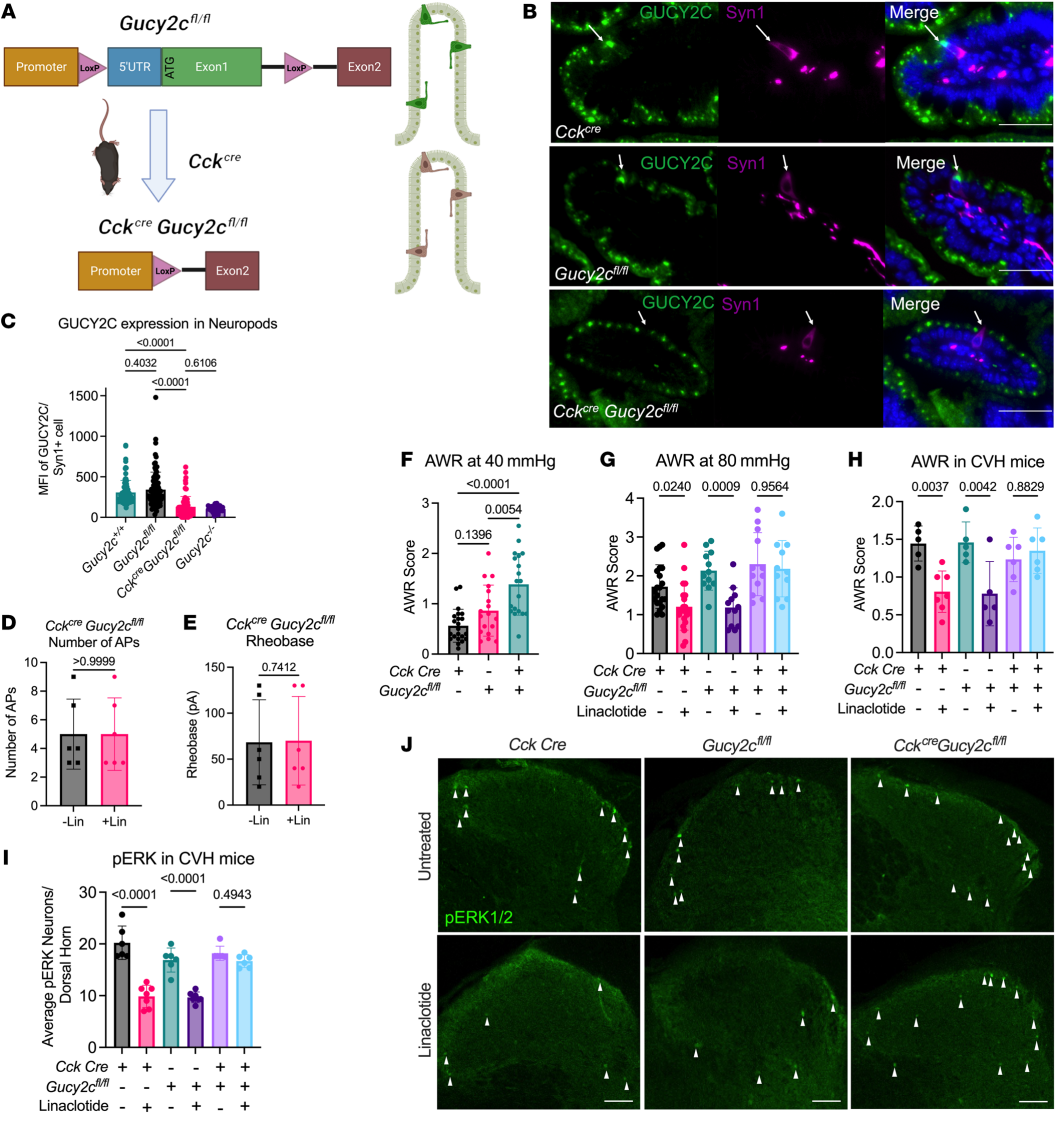
GUCY2C in intestinal neuropod cells regulates VP. (**A**) Schematic of *CCK^cre^Gucy2c^fl/fl^* mice used to eliminate GUCY2C in intestinal neuropod cells, with LoxP sites flanking Exon1 of the *Gucy2c* gene. (**B**) Representative immunofluorescence of *CCK^cre^*, *Gucy2c^fl/fl^*, and *CCK^cre^Gucy2c^fl/fl^* mice revealing GUCY2C^hi^ Syn1^+^ cells in *CCK^cre^*, *Gucy2c^fl/fl^* intestines, but loss of GUCY2C in *CCK^cre^Gucy2c^fl/fl^* Syn1^+^ cells (arrows; representative of n = 19–22). (**C**) Quantification of Mean Fluorescence Intensity (MFI) of GUCY2C in Syn1^+^ neuropod cells in intestine reveal that *CCK^cre^Gucy2c^fl/fl^* mice lose GUCY2C expression in neuropod cells (n = 3 mice, 10 sections/mouse, MFI/cell shown). (**D** and **E**) Electrophysiology of DRG neurons cocultured with intestinal crypts of *Cck^cre^Gucy2c^fl/fl^* mice show no change in (**D**) Rheobase and (**E**) AP number after linaclotide treatment (similar to *Gucy2c^–/–^*) (n = 6). (**F**) AWR scores of *Cck^cre^*, *Gucy2c^fl/fl^*, and *Cck^cre^Gucy2c^fl/fl^* mice at nonnoxious (40 mmHg) CRD reveals that *Cck^cre^Gucy2c^fl/fl^* mice recapitulate the visceral hypersensitivity observed in *Gucy2c^–/–^* mice (n = 19–22). (**G**) AWR scores of *Cck^cre^*, *Gucy2c^fl/fl^*, and *Cck^cre^Gucy2c^fl/fl^* mice after 80 mmHg CRD and the same mice after 4 μg/kg/day linaclotide treatment for 5 days (+Lin) reveal that *Cck^cre^* and *Gucy2c^fl/fl^*, but not *Cck^cre^Gucy2c^fl/fl^* mice, exhibit decreased pain scores after linaclotide (n = 11–21). (**H**) AWR scores of *Cck^cre^*, *Gucy2c^fl/fl^*, and *Cck^cre^Gucy2c^fl/fl^* mice after induction of CVH and 40 mmHg CRD before and after 4 μg/kg/day linaclotide treatment for 5 days (+Lin) reveals that *Cck^cre^* and *Gucy2c^fl/fl^*, but not *Cck^cre^Gucy2c^fl/fl^* mice, respond to linaclotide (n = 5–7). (**I**) pERK^+^ neurons in the dorsal horn of the spinal cord are decreased in CVH *Cck^cre^* and *Gucy2c^fl/fl^*, but not *Cck^cre^Gucy2c^fl/fl^*, mice after linaclotide (n = 6–7). (**J**) Representative images from **I**, showing decreased pERK staining in the dorsal horn of linaclotide-treated *Cck^cre^* and *Gucy2c^fl/fl^* mice, but not *Cck^cre^Gucy2c^fl/fl^* mice (arrowheads). Scale bars: 50 μm. Statistics for **C** and **F**–**I** were calculated using 2-way ANOVA with Tukey’s multiple comparisons test. Statistics for **D** and **E** were calculated using 2-tailed unpaired *t* test with an *f* test for comparison of variances.

The role of GUCY2C in regulation of VP in healthy mice and the development of a spontaneous VP syndrome in *Gucy2c^–/–^* mice suggest that neuropod-cell GUCY2C selectively controls visceral nociception. This hypothesis was explored by developing, for the first time to our knowledge, *Gucy2c^fl/fl^* mice, and placing GUCY2C under the control of *Cck^cre^*. This model eliminated GUCY2C expression selectively in CCK-expressing neuropod cells, but not in bulk intestinal epithelial cells (Figure 7, A–C). *Cck^cre^-Gucy2c^fl/fl^* intestines retained their ability to secrete extracellular cGMP with linaclotide stimulation, implying that extracellular cGMP release from the bulk intestine did not mediate the effect of linaclotide on VP- and DRG-neuron excitability (Figure 7, D and E, and Supplemental Figure 10). Importantly, *Cck^cre^-Gucy2c^fl/fl^* mice exhibited a spontaneous VP syndrome (Figure 7F) reminiscent of GUCY2C-deficient mice (Figure 6A). Moreover, like GUCY2C-deficient mice (Figure 6, E and F), *Cck^cre^-Gucy2c^fl/fl^* mice are unresponsive to GUCY2C agonist-induced visceral analgesia, and linaclotide did not decrease AWR pain scores or pERK staining in the dorsal horn (Figure 7, G–J). Together, these observations reveal that neuropod cell GUCY2C selectively controlled visceral nociception — rather than bulk intestinal epithelial cells — independent of secreted cGMP.

## Discussion

Here, we reveal that GUCY2C regulation of VP is mediated specifically by neuropod cells. A rare pool (< 1% of epithelial cells) of EEC cells concentrated in the small intestine were shown to overexpress GUCY2C by immunofluorescence, a *Gucy2c* promoter-driven GFP reporter, quantitative real-time PCR (qRT-PCR), RNA-Seq, and agonist-induced cGMP production. Their transcriptomic analysis demonstrates a gene-expression profile with characteristics of both EECs and neurons, but not of bulk epithelial cells. In cocultures, these cells produce DRG-neuron hyperexcitability when GUCY2C is unstimulated (or is off), but return excitability to baseline when GUCY2C in neuropod cells is stimulated by linaclotide (or is on), effects not recapitulated by bulk epithelial cells or extracellular cGMP. Consistent with this, linaclotide relieves VP produced by CRD in normal mice in a GUCY2C-dependent fashion. Eliminating GUCY2C expression produced a spontaneous VP syndrome in mice that was identical to that produced by colorectal exposure to TNBS. Of significance, selectively silencing GUCY2C expression in neuropod cells eliminated linaclotide regulation of neuron excitability and analgesia. These observations suggest a hypothesis in which GUCY2C expressed by neuropod, but not enterocyte, cells in the small intestine modulated afferent neuron excitability to regulate spinal-cord processing of visceral nociceptive signals ascending from the distal colorectum.

Results here align with emerging evidence suggesting that intestinal epithelial cells directly transmit nociceptive signals to innervating neurons. Optogenetic studies reveal bidirectional modulation of colon afferent nerves by intestinal epithelial cells that increase afferent spiking by activating epithelial channel rhodopsin (ChR2) and decrease spiking by inhibiting epithelial activation through archaerhodopsin (39, 40). These studies expressed light-sensitive channels, driven by Vil^cre^, in all intestinal epithelial cell types and could not discriminate contributions of specific cell types. In that context, ChR2 expression, driven specifically by *Cck^cre^* in neuropod cells, increased firing of vagal nodose ganglion neurons when photostimulated (28). Conversely, expressing the inhibitory light-sensitive channel Halo in neuropod cells, decreased nodose ganglion firing. Moreover, enterochromaffin cells (ECs), another EEC subtype that expresses chromogranin A, serotonin, and synapsin 1, directly transmit noxious luminal signals to closely associated 5-HT_3_R-expressing colonic afferent nociceptors (24). These observations support the hypothesis that neuropod cells could regulate visceral afferent neuron excitability and suggest a role for GUCY2C in regulating those gut-nervous system connections.

Previous studies suggest that intestinal epithelial cells secrete cGMP into the extracellular space in the submucosa, directly acting on an undefined extracellular target on peripheral afferent neurons, decreasing excitability. In mice, inhibition of MRP4, the ATP cassette anion transporter that mediates cGMP efflux from cells, blocks analgesia by GUCY2C agonists (19, 21). Importantly, extracellular, but not intracellular, cGMP dampens the firing of isolated, but not cocultured, mouse and human DRG neurons in vitro (22). Those observations notwithstanding, definitive in vivo studies demonstrated that lumenal application of GUCY2C agonists induces secretion of cGMP into the lumenal fluid, but not the submucosa. Indeed, cGMP levels quantified in situ in mouse intestinal submucosa by equilibrium dialysis — approximately 100 fmoles (41) — is approximately 4 orders of magnitude lower than levels that modulate afferent neuron firing — approximately 2 nmoles (19–23). Here, we demonstrate that extracellular cGMP is without effect on neuropod cell–induced DRG neuron hyperexcitability and does not recapitulate the effects of the GUCY2C-agonist linaclotide.

Beyond extracellular cGMP, the precise mechanisms by which neuropod cell GUCY2C regulates neuronal excitability remain to be defined. Although enteroendocrine hormones are expressed by GUCY2C^hi^ cells, their secretion into blood was not altered in *Gucy2c^–/–^* mice (Supplemental Figure 11), making it unlikely that the effects of GUCY2C on VP were mediated by those hormones. Neuropod cells communicate with neurons through canonical neurotransmitters (e.g., glutamate) (28, 33). Analysis of neurotransmitter gene expression reveals that GUCY2C^hi^ cells differentially express machinery for monoamine synthesis and processing (Supplemental Figure 12). EECs stimulated by noxious agonists release the monoamine serotonin which stimulates 5-HT_3_R-expressing nociceptive afferents (24). In that context, GUCY2C^hi^ cells most highly express genes involved in serotonin production and packaging (*Tph1, Slc18a1,* and *Slc18a2*), suggesting that they also might regulate nociceptive signaling by DRG neurons through serotonin (24). Further exploration of the mediator between GUCY2C^hi^ neuropod cells and DRG neurotransmission are essential to our understanding of their interaction.

Previous observations suggest that colorectal nociceptive afferents can be regulated by local activation of GUCY2C following TNBS sensitization. Thus, linaclotide was instilled directly into the colon by enema minutes before colorectal distention, leading to decreased visceromotor responses to pain in rodents with visceral hypersensitivity (19). However, it is unlikely that the effects of linaclotide delivered by enema to the colon mediate the activity of oral GUCY2C ligands in healthy mice or humans. Linaclotide has a chymotrypsin cleavage site mediating proteolytic destruction in the proximal small intestine, resulting in less than 1% of the administered linaclotide having been recovered in stool (42). In close agreement, super-therapeutic doses (870 μg/day × 7 days) of oral linaclotide fail to activate GUCY2C signaling in the colorectum in humans (43). Similarly, plecanatide is maximally active in the acidic environment of the proximal duodenum, without obvious activity in the colorectum (43, 44). These data suggest that oral GUCY2C agonists relieve VP produced in the rectum by activating GUCY2C in small intestine. This suggestion is supported by the observations that GUCY2C^hi^ neuropod cells are more plentiful in small intestine (Supplemental Figure 1) and that oral linaclotide activates phosphorylation of the downstream cGMP–target VASP in the small intestine in these models (Supplemental Figure 13). Together, these observations suggest that oral GUCY2C ligands relieve colorectal pain by modulating nociceptive afferent signals in the spinal cord originating from anatomically distal sites.

While circuits mediating regulation of pain from the colorectum by linaclotide stimulation of GUCY2C in small intestine neuropod cells remains to be defined, there is precedent for this type of cross-talk. Viscero-visceral inhibition and sensitization has been described between abdominal and pelvic organs, including the small intestine and colon (45–47). Indeed, distention of the jejunum directly inhibits visceromotor responses induced by colonic distention (48). This crosstalk between these organs has been attributed to dual innervating afferents comprising DRG neurons with axonal projections that innervate 2 distal organs (21, 22). Oral linaclotide could modulate these dual-innervating afferents by stimulating GUCY2C in neuropod cells in the small intestine to reduce VP signaling from colorectal distention, as well as from bladder irritation and endometriosis (22, 49). In that context, only formulations of linaclotide targeted to the small intestine, but not the colon, relieve VP in patients with IBS (50, 51). Beyond circuits connecting the proximal and distal intestine, a role for GUCY2C in neuropod cells in modulating redundant VP pathways and pain modalities beyond mechanical distention (e.g., thermal and toxic) remain to be explored (52, 53).

In summary, GUCY2C is highly expressed in neuropod cells concentrated in the small intestine. GUCY2C^hi^ neuropod cells interact closely with subjacent neurons and modulate their excitability. Indeed, silencing GUCY2C produces neuron hyperexcitability, with a reduced rheobase and increased AP firing. Conversely, inducing GUCY2C signaling in neuropod cells returns DRG-neuron excitability to basal levels, increasing rheobase and inhibiting repetitive AP firing. While the molecular basis of this regulation remains to be defined, it is not mediated by bulk epithelial cells or extracellular cGMP. In the absence of GUCY2C signaling, mice exhibit a spontaneous VP syndrome that recapitulates TNBS postinflammatory sensitization. Further, activation of GUCY2C signaling by oral linaclotide, which is confined to the small intestine, relieves VP produced by CRD in normal mice. Importantly, linaclotide-dependent analgesia is specifically mediated by neuropod cells, and elimination of GUCY2C expression in those cells in *Cck^cre^Gucy2c^fl/fl^* mice produces insensitivity to linaclotide-induced regulation of neuron excitability and analgesia.

These studies expand the mechanistic and translational dimensions of GUCY2C as a therapeutic target for VP. They identify a role of GUCY2C in neuropod cells in regulating afferent neuron excitability and visceral analgesia. Gene expression profiling of these cells offers unique opportunities to tailor molecularly targeted approaches to amplify GUCY2C signaling selectively in neuropod, but not bulk epithelial, cells to maximize analgesia and minimize secretory side effects. Indeed, transcriptomic profiling revealed the differential expression of important phosphodiesterases (PDEs) that degrade cGMP in GUCY2C^hi^ and GUCY2C^med^ cells (see Figure 2B). In that context, the differential expression of PDE5 in GUCY2C^med^ cells, but PDE3 in GUCY2C^hi^ cells, offers an opportunity to use isotype-selective PDE inhibitors, in conjunction with linaclotide or other GUCY2C agonists, to amplify cGMP signaling specifically in neuropod cells that drive visceral analgesia, but not in enterocytes that mediate intestinal secretion and diarrhea. Ultimately, translation of these findings into combinatorial therapeutics could provide patients with VP options for analgesia without opioids, with maximum clinical efficacy, and without unwanted side effects. Moreover, beyond pain associated with chronic constipation syndromes, mechanisms here likely mediate the analgesic effects of GUCY2C agonists on VP associated with chronic bladder dysfunction and endometriosis (22, 49).

## Methods

### Mouse studies.

Mice for these studies were bred, maintained, genotyped, and functionally characterized in the animal care facility at Thomas Jefferson University. *Gucy2c^–/–^* mice on a C57Bl6/J background were maintained within our colony (54–58). *Gucy2c^fl/fl^* mice were developed in conjunction with the CRISPR-Cas9 Mouse Targeting Core at University of Pennsylvania (RRID:SCR_022378). *Gucy2c-GFP* mice [RRID: MMRRC_030480-UCD] were developed by the GENSAT project and purchased from the Mutant Mouse Research and Resources Center. These mice were maintained on a CD1 (Crl:CD1(ICR)) background purchased from Charles River Laboratories. *Cck^cre^* (Stock: 012706) and *tdTomato^fl/fl^*(Stock: 007914), were purchased from the Jackson Laboratory. *Neurog3^cre^* mice were generously provided by Ingolf Bach, University of Massachusetts (Worcester, MA) and developed in the laboratory of Andrew Leiter, University of Massachusetts (59). Crosses of *Gucy2c-GFP* mice with *tdTomato^fl/fl^* mice were performed so that experimental mice were 50% C57Bl6/J and 50% CD1 — using only the first cross. Mice were raised with 12 hour light-12 hour dark cycles and were used from age 4–30 weeks unless otherwise indicated. All mice were compared with littermate controls or bred as F2 crosses of *Gucy2c^+/+^* and *Gucy2c^–/–^* from heterozygous parents.

### Intestinal immunofluorescence.

Intestines were prepared as previously described (54, 60). Briefly, the intestines were isolated from freshly sacrificed mice and flushed with PBS using a 20 G needle and a 20 mL syringe. Intestines were opened longitudinally, fixed in 4% paraformaldehyde (PFA) (Thermo Fisher Scientific) overnight, Swiss rolled, and stored in 70% Ethanol for up to 1 week. Samples were then paraffin embedded and sectioned at 4 μM sections onto slides. After deparaffinization and rehydration, sections underwent antigen retrieval with pH 9 Dako Antigen Retrieval Solution (Agilent Technologies) in a pressure cooker for 15 minutes under high pressure. Samples were then blocked for 1 hour in blocking buffer — either 10% milk in PBS with 0.3% Triton-X or 5% BSA in PBS with 0.3% Triton-X for phospho-proteins. Slides were then incubated in primary-antibody solution (diluted in blocking buffer) overnight at 4 °C, washed 3 times in PBS with 0.1% Tween-20 (PBST), and incubated in blocking buffer with secondary antibody and nuclear counterstain DAPI for 60 minutes at room temperature. For tyramide amplification of GUCY2C, samples were washed 3 times in PBST and incubated for 10 minutes in house-made tyramide FITC at a final concentration of 100 μg/mL in PBS with 0.003% H_2_O_2_ (58, 61). Following a final series of washes, samples were mounted onto slides with coverslips with Prolong Diamond Antifade mounting media (Thermo Fisher Scientific). Antibodies can be found in Supplemental Table 1.

### Small intestinal dissociation and FACS sorting.

Mice were sacrificed by cervical dislocation and the small intestines were flushed in cold PBS, cut longitudinally, and incubated in dissociation buffer made of DMEM without Ca^2+^ and Mg^2+^ (Corning), 10 mM EDTA (Invitrogen), 10% FBS (Cytiva) for 1 hour, rotating at 4°C. After shaking, vortexing, and centrifugation, the intact muscularis layer was removed and dissociated epithelium remained in suspension. The epithelium was then divided into 2 tubes and each tube was dissociated in 5 mL of 0.3 U/mL Dispase II (Sigma-Aldrich) for 15 minutes at 37°C, shaking every 5 minutes. Cells in Dispase II solution were spun down at 500*g* for 5 minutes at 4°C, then resuspended in 10 mL Neurobasal+ medium. Neurobasal+ medium consists of Neurobasal A (Gibco), B-27 (Invitrogen), N-2 (Gibco), Y-27632 rock inhibitor (StemCell Technologies), NGF (Peprotech), artemin (Peprotech), Antibiotic-Antimycotic (Gibco), and Glutamax (Gibco). To decrease clumping and increase sort rate, 50 μL of 2000 U/mL DNAse I (Grade II; Roche) was added to every 1 mL of single-cell suspension. This solution was filtered through a 70 μm filter, and single cells were counted. Cells were then prepared for FACS at a concentration of 3 million cells/mL and live/dead stained with 1 μL/mL of Sytox Red (Thermo Fisher Scientific). Cells were then sorted on a BD Melody (BD Biosciences). Debris, doublets, and dead cells were gated out, leaving a population of live, single cells that were sorted based on fluorescence intensity.

### mRNA isolation and qRT-PCR.

mRNA isolation was performed on 20,000 cells/sample isolated by cell sorting on the BD Melody, detailed above. For each experiment, at least 3 mice were used (n = 3), with at least 3 biological and 2 technical replicates per mouse per qRT-PCR plate. mRNA was isolated using the RNeasy Plus Micro kit (Qiagen). Cells were sorted directly into 0.35 mL RLT Plus buffer, vortexed, and frozen at –80°C for further processing. Reverse transcription was performed with TaqMan Reverse Transcription Reagents (Thermo Fisher Scientific) according to manufacturer’s instructions. qRT-PCR was performed using Applied Biosystems TaqMan Master Mix (Thermo Fisher Scientific); Taqman Primer Probes are listed in Supplemental Table 2. Relative expression was calculated with *Gapdh* as the reference gene for all samples and as 2^–ΔΔCt^.

### RNA-Seq.

RNA-Seq was performed on RNA isolated as above using the RNAeasy Plus Micro Kit. Five 10-week-old litter-matched *Gucy2c-GFP* mice were sorted into GUCY2C^hi^ and GUCY2C^Med^ groups at 20,000 cells per group. Library preparation, sequencing, alignment, and read counting was performed by the Novogene Corporation (Tianjin, China). Libraries were prepared using NEBNext Ultra II RNA Library Prep Kit (New England BioLabs). More information on library preparation and sequencing can be found in the Supplemental Methods.

Subsequent RNA sequencing analysis was performed in R (R Foundation for Statistical Computing). For analysis, only genes with counts per million (cpm) of greater than 0.5 in at least 3 of the samples were included (15,689 genes). Limma (v3.40.6) and edgeR (v3.26.8) packages were used to determine differential gene expression (adj P value < 0.01) (62, 63). GSEA was performed using the GSEA (v4.1.0) C8 collection of cell type–signature gene sets (64, 65). Upregulated genes were ranked by log-fold change, and GSEA results were plotted using Prism (v9.3.1). Over-represented synaptic terms were analyzed using the online portal for SynGO, comparing upregulated genes in GUCY2C^hi^ cells (p adj > 0.01) to all genes with more than 0.5 cpm (31). Gene ontology (GO) analysis was performed using the package gProfileR version 0.70 (66). Data from RNA-Seq experiments can be acquired through the NCBI Gene Expression Omnibus (GEO; GEO Accession number: GSE207870).

### Data set acquisition and analysis.

Human single-cell RNA data was downloaded from the Human Protein Atlas on December 6, 2021 (38). Neurog3-Chrono mouse data was downloaded from GEO, data set ID GSE113561, on May 4, 2020 (36). GLP-1 in human organoid data was downloaded from GEO, data set ID GSE148224 (37). These publicly available data were analyzed with limma and edgeR packages (62, 63) in R, and *Gucy2c* FPKM was compared in Prism using a 1-way ANOVA.

### Coculture of intestinal cells with embryonic DRG neurons.

Intestinal cells used in coculture experiments were cultured as whole crypts to improve cell viability. As reported previously, yields of viable neuropod cells were low in sorted coculture, even when plating at a high density (27). Maintaining the intestinal epithelium as whole crypts improved the health of both neuropod cells and DRG neurons in coculture, allowing for more consistent cultures and more reproducible results. DRG neurons were prepared from E15.5 C57Bl6/J mice. Cocultures were grown on Matrigel-coated coverslips prepared by adding 20% Matrigel in DMEM to each coverslip and allowing the gel to solidify for at least 3 hours before plating. Neurobasal+ medium was used to culture intestinal cells with neurons, as this solution provided viable outgrowth conditions for both cell types.

Intestinal crypts were prepared as described in the small intestinal organoid culture section (Supplemental Methods) from *Gucy2c-GFP* mice. 100-200 crypts per well were plated directly onto Matrigel-coated coverslips simultaneously with isolated DRG neurons in a 24-well plate (Thermo Fisher, 144530).

Embryonic DRG neurons were isolated as described previously (67, 68). Briefly, DRGs were dissected out of E15.5 embryos on ice in L15 media (Gibco). Media was pipetted off, and DRGs were digested in 0.25% Trypsin (Corning) for 15 minutes at 37°C, gently shaking every 5 minutes. DRGs were then transferred to Neurobasal+ medium and mechanically triturated with a fire-polished glass pipette to achieve single-cell suspensions. DRG neurons were counted, and 5,000–10,000 neurons were plated per well with intestinal cells. Intestinal cells and DRG neurons were allowed to settle in 100 μL of Neurobasal+ medium on top of the coverslip for 30 minutes before adding the remaining 400 μL of media to each well. Live cocultures were imaged 24–48 hours after plating on an EVOS FL Auto Imaging system (Thermo Fisher Scientific). For immunostaining, cells were fixed with warm 4% PFA 24 hours after plating. DRG-neuron outgrowth to neuropod cells was quantified at 20× magnification in live cultures on D6 by selecting areas of the coverslip positive for GFP^hi^ cells in the GFP channel and taking a phase contrast image with a superimposed GFP image. All DRG neurons present in the image were counted, and subsequently, all DRG neurons with processes abutting GFP^hi^ cells were counted as “near neuropod cells” (n = 17 coverslips).

### Electrophysiology.

Electrophysiology experiments were performed on intestine-DRG neuron cocultures as prepared above 12–36 hours after plating. To patch on DRG neurons that were connected to GUCY2C^hi^ neuropod cells, first a GFP-bright neuropod cell was identified on the plate using GFP fluorescence. Next, the view was switched to brightfield, and a DRG neuron in physical contact (either on the soma or with a projection) was selected to patch. To identify DRG neurons alone, the processes of each DRG neuron were traced using DIC to ensure that the projections did not reach intestinal tissue.

Patch electrodes were made from Corning 7056 thin wall capillary glass (Warner Instruments) and pulled with a P-97 micropipette puller (Sutter Instruments). Electrodes were fire polished to have tip resistances of 1.5–4.5 MΩ. Signals were amplified using a Multiclamp 700B amplifier (Molecular Devices), low-pass filtered at 2 kHz (4-pole Bessel), digitized at 10 kHz using Digidata 1440 (Molecular Devices), and stored in a computer using Clampex version 10.2 software (Molecular Devices).

DRG neurons were recorded in the whole cell configuration. The external solution was composed of 130 mM NaCL, 5 mM KCl, 2 mM CaCl_2_, 1 mM MgCl_2_, and 10 mM HEPES, at pH 7.4 and titrated with NaOH. The internal solution was composed of 130 mM K-MES, 1 mM CaCl_2_, 1 mM EGTA, and 10 mM HEPES, at pH 7.3 and titrated with KOH, with 2 mM of Mg-ATP and 0.3 mM Tris-GTP added on the day of recording. All compounds used in the solutions were purchased from Sigma-Aldrich. The resting membrane potential was recorded in the absence of current injection. To determine the rheobase, the neurons received 200-millisecond current injection pulses starting at –40 pA from 0 pA baseline, which was then followed by consecutive current injection pulses increasing at intervals of +10 pA until the neuron fired an AP. To assess repetitive AP firing, neurons received a 1-second current injection pulse at 3× rheobase from a 0-pA baseline. Data processing and analysis were conducted in Clampfit version 10.5 (Molecular Devices) and Origin Pro version 9.1 (Origin Laboratory).

### Induced visceral hypersensitivity.

To induce visceral hypersensitivity, mice were treated with intrarectal TNBS (Sigma Aldrich). TNBS is commonly used to induce colitis and subsequent visceral hypersensitivity, resulting in hallmarks of visceral hypersensitivity including heightened pain responses to colorectal distention and increased sprouting and firing of nociceptive afferents that innervate the intestine (19, 22, 69, 70). Intrarectal TNBS induces acute colonic inflammation that resolves at 28 days, resulting in mice with chronic visceral hypersensitivity (CVH) that are sensitized to VP without active inflammation (71). Briefly, mice were fasted and anesthetized lightly with 2% isofluorane. A lubricated polyurethane catheter was inserted 2 cm intrarectally to instill 100 μL of 130 μL/ml TNBS in 30% ethanol into the intestine.

### Animal linaclotide treatment.

Linaclotide was diluted in sterile PBS at 0.5 μg/mL and administered by oral gavage daily for 5 days. Mice were given a 4 μg/kg dose of linaclotide — 200 μL of 0.5 μg/mL linaclotide — for allometric approximation of the FDA-approved 290 μg dose of linaclotide prescribed to patients (72, 73).

### CRD and pERK immunofluorescence.

CRD and pERK immunofluorescence was performed as previously described (19, 22, 69). Mice were fasted overnight to reduce stool content and anesthetized with 2% isofluorane. A lubricated 6 fr Foley catheter (Medline) was inserted transanally until the proximal end of the balloon was 0.5 cm from the anal verge, with a total balloon insertion of 52 cm, and secured with tape to the mouse’s tail (74). The catheter was then secured to the tail using tape, and the mouse was given 5 minutes to recover from anesthesia and acclimate to the balloon before CRD. CRD was performed using a Leur-lock 5 mL syringe (BD Biosciences) to deliver 80 mmHg of pressure — 1.2 mL of air per plunge — as assessed with a custom sphygmomanometer apparatus. The balloon was inflated to 80 mmHg for 10 seconds, followed by 5 seconds of deflation, and repeated 5 times. The mouse was immediately i.p. injected with 1 ug/kg avertin anesthesia for perfusion. Once the anesthetized mice were unresponsive to a toe pinch, mice were perfused with ice-cold PBS and ice-cold 4% PFA. The spinal cord from T10–L1 was removed, using ribs and the lumbar enlargement as landmarks, and postfixed in 4% PFA at 4°C for 16 hours, moved to 0.1% phosphate buffer for 24 hours, then submerged in 30% sucrose for 48 hours. Spinal cords were frozen in Tissue-Tek O.C.T. Compound (Sakura Finetek) and sectioned at 30 μm onto Superfrost Plus Microscope Slides (Thermo Fisher Scientific). Five spinal cord sections were sectioned per slide, and each slide was numbered so that equivalent position across the thoracolumbar spinal cord was compared for each condition. Slides were allowed to dry at room temperature overnight, then were frozen at –20°C.

To visualize pERK, slides were incubated at 37°C for 1 hour, and O.C.T. Compound was washed off with PBST. Tissue was outlined on slides with a Super PAP Pen (Thermo Fisher Scientific) and blocked for 1 hour in 5% BSA diluted in PBS with 0.3% Triton-X (PBSTx). Tissue was then incubated with pERK antibody at 1:200 in 5% BSA PBSTx for 16 hours at room temperature in a humid chamber. Rabbit control IgG was used as a negative control during this step at an equivalent concentration to pERK (2.5 μg/mL blocking buffer). Slides were then washed 3 times in PBST and incubated with Alexa Fluor 488-donkey-anti-rabbit antibody and DAPI (1:5000) in blocking buffer for 1 hour at room temperature. Slides were then washed 3 times in PBST and mounted with Prolong Diamond Antifade mounting media. For each condition, all genotypes were stained together (*Gucy2c^+/+^* and*Gucy2c^–/–^* or *CCK^cre^*, *Gucy2c^fl/fl^*, and *CCK^cre^Gucy2c^fl/fl^*). Slides were compared with negative-control slides stained with a rabbit IgG isotype control primary antibody.

Slides were imaged on a Leica TCS SP8 SMD Microscope (Jefferson Neuroimaging Core). All slides were imaged at the same settings, and for each condition all genotypes were imaged together. For all sections, z-stacks through all 30 μm were used with a z-step of 5 μm. Sections were imaged at 10× magnification, and the full z-stack (30 μm) of least 3 sections were counted per mouse. Counting was performed manually by a blinded observer, where a pERK-positive neuron was defined as having fluorescence above IgG control and being present in more than 1 consecutive z-step.

### AWR.

Behavioral responses of mice to CRD were quantified by the AWR (74–76). All mice used for AWR were at least 12-weeks old to account for mice that were given TNBS at 8 weeks. Mice were fasted, anesthetized, and a catheter was placed in the same manner as CRD for pERK experiments above. Mice then underwent either a 40 mmHg CRD protocol or an 80 mmHg CRD protocol. For the 40 mmHg protocol, a 1 mL Luer-Lok Tuberculin Syringe (BD Biosciences) was used to inject 0.6 mL of air, while the 80 mmHg protocol used a 5 mL Leur Lok syringe with 1.2 mL of air, with pressures measured by a sphygmomanometer. In the 40 mmHg protocol, the balloon was inflated for 2 seconds every 30 seconds, 10 times for each mouse; and for 80 mmHg, the balloon was inflated 2 seconds every 30 seconds 5 times for each mouse. Mice were scored on a scale from 0–4 (77, 78) with an AWR of 0 indicating lack of response; AWR 1 for brief movement resulting in immobility, or beginning mobility; AWR 2 for contraction of abdomen without lifting of abdominal structure; AWR 3 for lifting of abdominal structure with arching of back; AWR 4 for lifting of pelvic and abdominal structures with marked extension of limbs and arching.

All mouse behavior was scored by a blinded observer and video was recorded for each mouse for confirmatory scoring. The average of all 5 or 10 AWR scores per mouse was used for final statistical analysis. For mice that were tested more than once (i.e. with and without linaclotide), CRD protocols were spaced at least 4 weeks apart, allowing for adequate recovery between CRD protocols. For each condition, relevant genotypes were scored together on the same day, with *Gucy2c^+/+^* and *Gucy2c^–/–^*together, and *Cck^cre^*, *Gucy2c^fl/fl^*, and *Cck^cre^Gucy2c^fl/fl^* together.

### VMR.

Electromyographic (physiological) responses of mice to CRD were quantified by the VMR.(79, 80) Briefly, mice were anesthetized with 1.2–1.4 g/Kg of urethane (Sigma-Aldrich) through i.p. injection. After 20 minutes, mice were shaved and further anesthetized with 1.5% isoflurane to dissect the external oblique muscle. Bipolar electrodes (Micrograbber test clips [Pomona Electronics]) spaced 3 mm apart were placed along the muscle fiber of the external oblique. A needle electrode was placed subcutaneously in the tail of the mouse as a ground. Isoflurane was stopped and mice were allowed to recover until visibly responsive to a hind-paw toe pinch with forceps. VMR activity was measured using an AD Systems BioAmp. The VMR to CRD was continuously recorded at least 1 minute before, as a baseline, and after CRD, performed manually with 80 mmHg of pressure, for 10 seconds every 4 minutes and repeated 5 times total per animal. The VMR was amplified (1,000 × 3),filtered through a band-pass filter (0.3–1.0 kHz bandpass), sampled at 2.0 kHz using a PowerLab 8/30 data acquisition system (AD Instruments). VMR recordings were then rectified and integrated, with time constant decay of 0.05 seconds, using LabChart 7 software (AD Instruments). The AUC was averaged for 10 seconds before stimulation, and this background was subtracted from the measured AUC after stimulation.

### Data availability.

Data from RNA-Seq experiments were deposited in the NCBI Gene Expression Omnibus (GEO Accession number: GSE207870).

### Statistics.

Results are presented as the mean ± SD, and a P value of < 0.05 was considered significant. Statistical analysis was performed in Graphpad Prism 9 (Version 9.3.1) unless otherwise stated. Data were analyzed via 1-way and 2-way ANOVAs with Tukey’s multiple comparisons tests and Šídák’s multiple comparisons tests, as well as 2-tailed unpaired Student’s *t* tests with *f* tests to compare variances. Grubb’s outlier test was conducted on all AWR data. Plots were generated using limma and edgeR in R.

### Study approval.

The Thomas Jefferson University IACUC approved all animal protocols and procedures under protocol 01357. Human duodenal specimens were obtained from Whipple procedures performed for pancreatic cancer resection at Thomas Jefferson University Hospital. Samples were deidentified before retrieval, and immediately fixed overnight in 4% PFA for immunofluorescence. Although patients undergoing Whipple procedures had pancreatic cancer, their duodena were used to visualize the presence of neuropod cells in the human small intestine. Functional studies were not performed on these samples.

## Author contributions

These investigations were conducted, supporting methodologies developed, and data were acquired and validated by JRB, AKL, TDA, AAE, and SB. Data were visualized and the original draft was prepared by JRB. The project was conceptualized, data were reviewed, and the manuscript was reviewed and edited by JRB, AKL, TDA, AAE, SB, LC, ACL, AES, MC, and SAW. SAW provided administration and acquisition of financial support for the project leading to this publication.

## Supplementary Material

Supplemental data

Supplemental data set 1

## Version 1 12/22/2022

In-Press Preview

## Version 2 02/15/2023

Electronic publication

## Version 3 08/22/2023

Annie K. Londregan's name was corrected in the author list.
