# Supplementary material for: Intestinal neuropod cell GUCY2C regulates visceral pain
Source: J Clin Invest. 2023 Feb 15;133(4):e165578. doi: 10.1172/JCI165578 (PMC9927949; doi:10.1172/JCI165578)
Supplement: Supplemental data [file jci-133-165578-s008.pdf]

Supplemental Figures

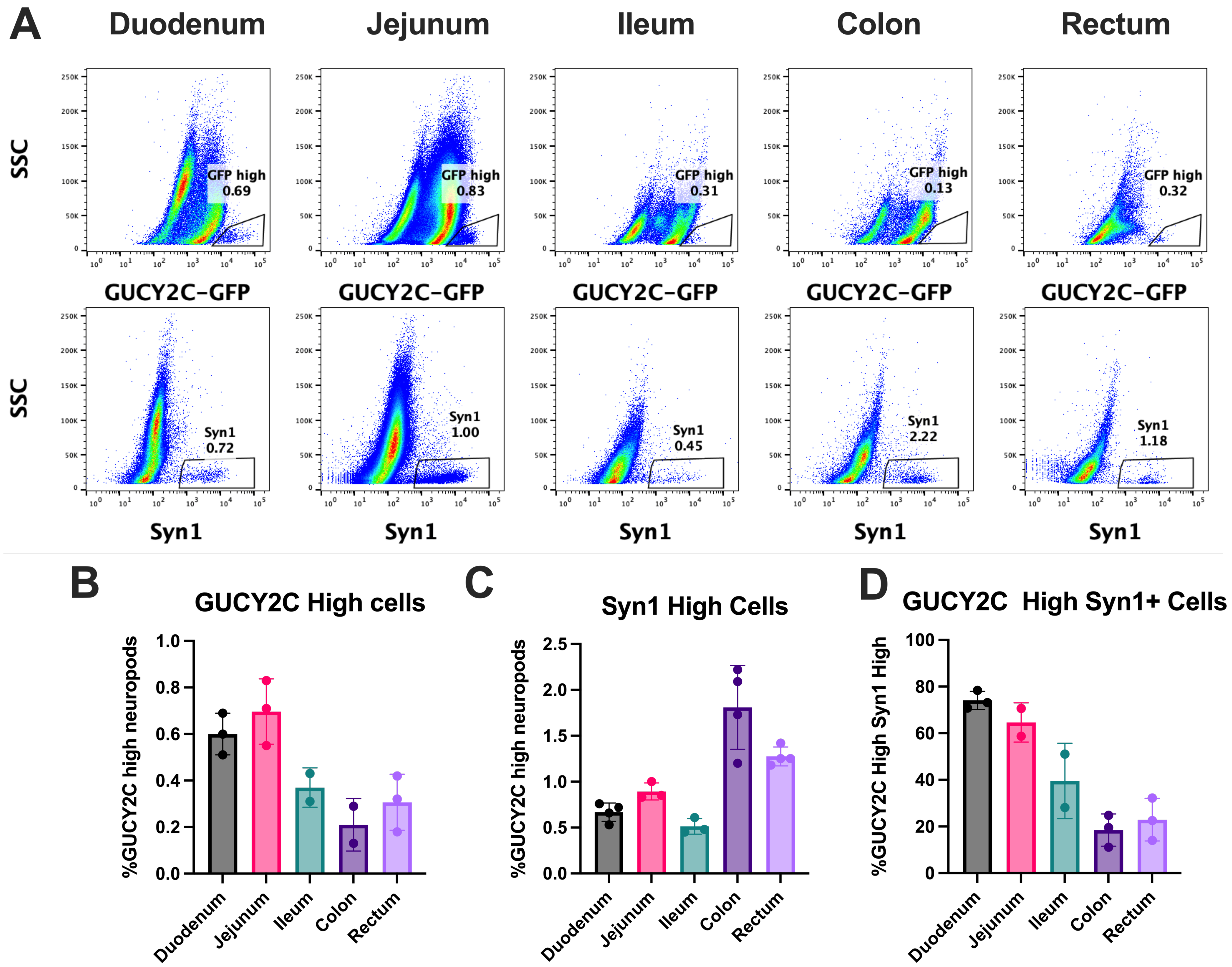

Supplemental Figure 1. GUCY2C^{High} cells are enriched in the proximal small intestine. A. Representative flow cytometry plots of dissociated *GUCY2C-GFP* mouse intestine. Anatomical sections of the intestine were dissociated separately and stained intracellularly with Syn1. **B.** Percentage of GUCY2C^{High} neuropod cells present in the bulk epithelium in each intestinal region. **C.** Percentage of Syn1 High cells present in the bulk epithelium in each intestinal region. **D.** Percentage of Syn1 High cells that are also GUCY2C^{High} per intestinal region shows that GUCY2C^{High} neuropod cells are enriched in Duodenum and relatively sparse in Colon and Rectum. (n=2-4 mice per group, Data plotted as mean ± SD).

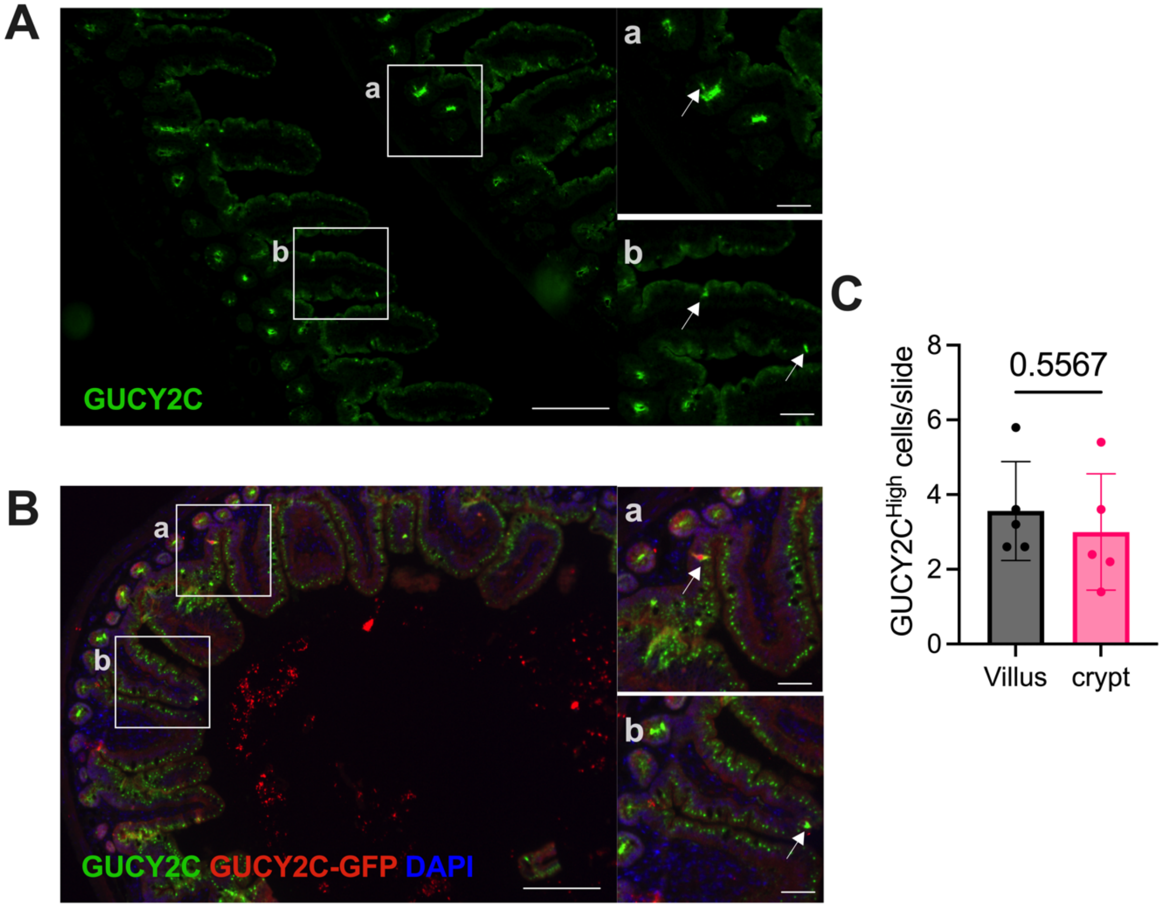

Supplemental Figure 2. GUCY2C^{High} cells are equally distributed across the crypt-villus axis. **A-B.** Immunofluorescence of GUCY2C in representative image of mouse small intestine reveals GUCY2C^{High} cells (arrows) in crypts (**a**) and villi (**b**). **C.** Quantification of GUCY2C high cells/image (n=5 mice, 5 images/mouse) shows that GUCY2C^{High} cells are present equally in crypt and villus. Scale bar in A-B = 100 µm, scale bar in a-b = 50 µm.

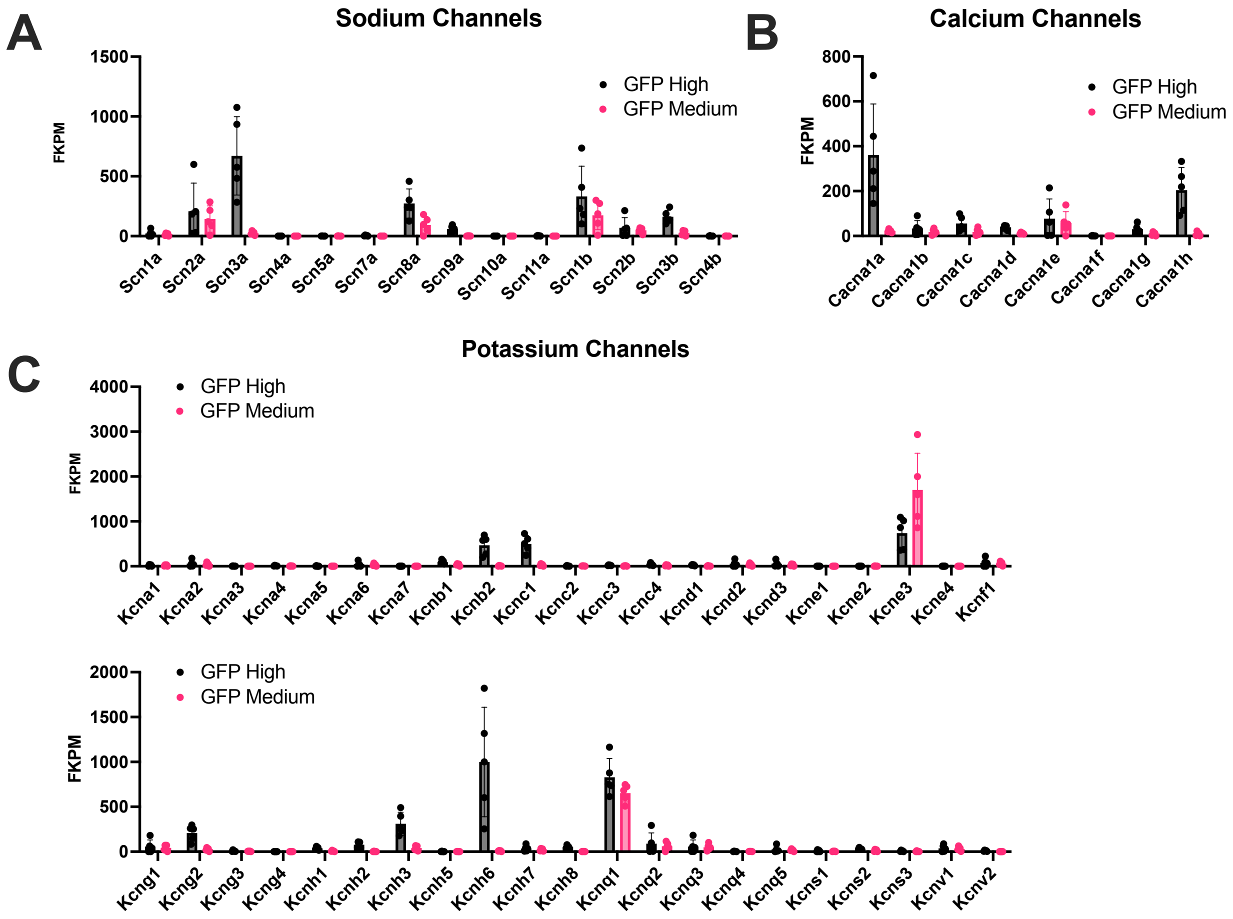

Supplemental Figure 3. GUCY2C-GFP^High cells differentially express voltage-gated ion channels. RNAseq data comparing GUCY2C-GFP^High and GUCY2C-GFP^Medium intestinal epithelium shows differential transcriptional expression of **A.** Voltage gated sodium channels **B.** Voltage gated calcium channels **C and D.** Voltage gated potassium channels (n=5 mice).

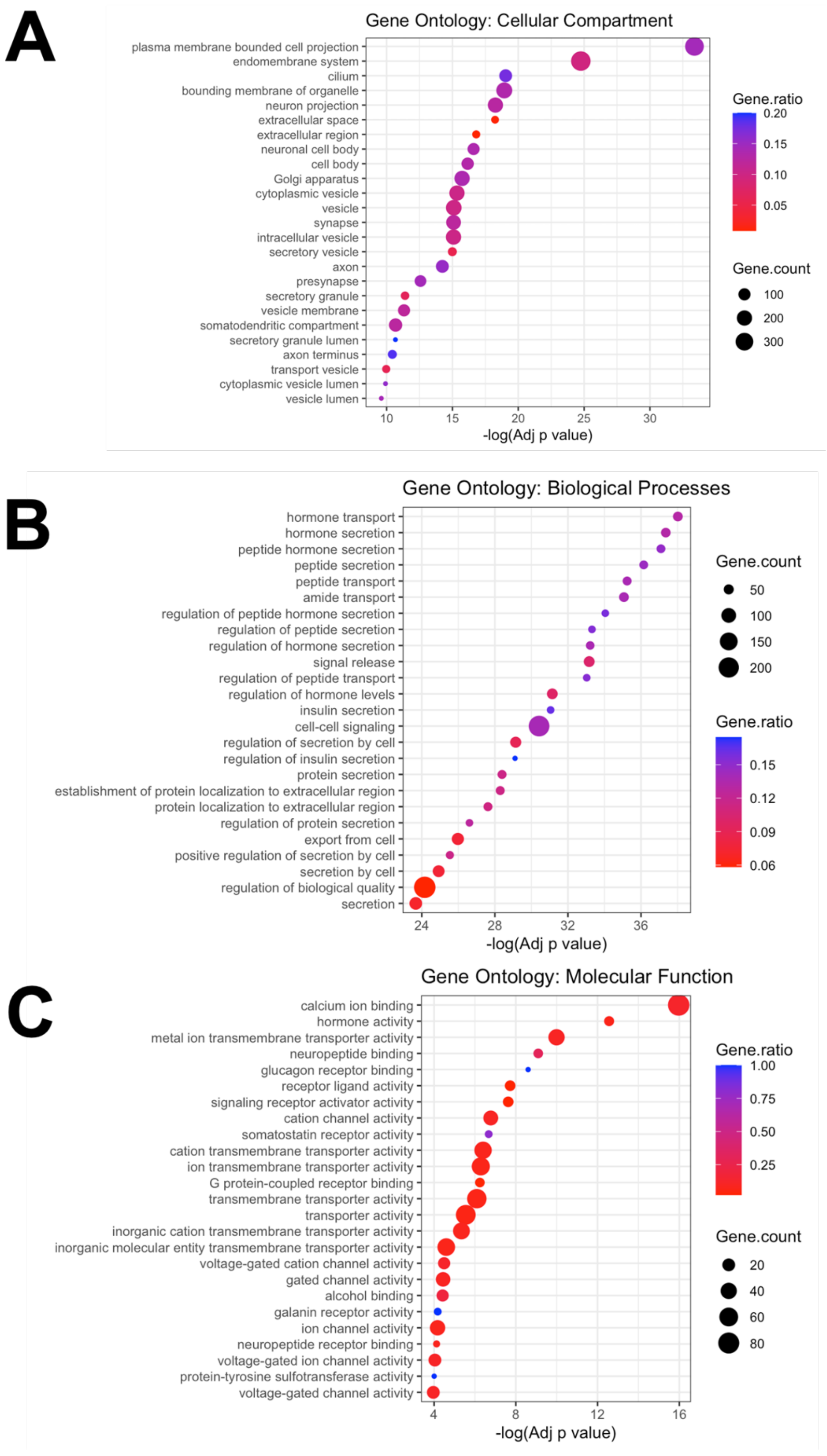
A
Gene Ontology: Cellular Compartment
plasma membrane bounded cell projection
endomembrane system
cilium
bounding membrane of organelle
neuron projection
extracellular space
extracellular region
neuronal cell body
cell body
Golgi apparatus
cytoplasmic vesicle
vesicle
synapse
intracellular vesicle
secretory vesicle
axon
presynapse
secretory granule
vesicle membrane
somatodendritic compartment
secretory granule lumen
axon terminus
transport vesicle
cytoplasmic vesicle lumen
vesicle lumen
10 15 20 25 30
-log(Adj p value)
Gene.ratio
0.20
0.15
0.10
0.05
Gene.count
100
200
300
B
Gene Ontology: Biological Processes
hormone transport
hormone secretion
peptide hormone secretion
peptide secretion
peptide transport
amide transport
regulation of peptide hormone secretion
regulation of peptide secretion
regulation of hormone secretion
signal release
regulation of peptide transport
regulation of hormone levels
insulin secretion
cell-cell signaling
regulation of secretion by cell
regulation of insulin secretion
protein secretion
establishment of protein localization to extracellular region
protein localization to extracellular region
regulation of protein secretion
export from cell
positive regulation of secretion by cell
secretion by cell
regulation of biological quality
secretion
24 28 32 36
-log(Adj p value)
Gene.count
50
100
150
200
Gene.ratio
0.15
0.12
0.09
0.06
C
Gene Ontology: Molecular Function
calcium ion binding
hormone activity
metal ion transmembrane transporter activity
neuropeptide binding
glucagon receptor binding
receptor ligand activity
signaling receptor activator activity
cation channel activity
somatostatin receptor activity
cation transmembrane transporter activity
ion transmembrane transporter activity
G protein-coupled receptor binding
transmembrane transporter activity
transporter activity
inorganic cation transmembrane transporter activity
inorganic molecular entity transmembrane transporter activity
voltage-gated cation channel activity
gated channel activity
alcohol binding
galanin receptor activity
ion channel activity
neuropeptide receptor binding
voltage-gated ion channel activity
protein-tyrosine sulfotransferase activity
voltage-gated channel activity
4 8 12 16
-log(Adj p value)
Gene.ratio
1.00
0.75
0.50
0.25
Gene.count
20
40
60
80

Supplemental Figure 4. Gene Ontology of GUCY2C^{High} cells reveals increased expression of pseudopods, hormone and signal release, endocrine, and neuronal function. A. The most upregulated terms in cell compartment Gene Ontology shows that GUCY2C^{High} cells highly express genes related to cellular projection and neuronal projection (as evidenced by their unique "pseudopod" morphology), as well as vesicles, synapses, axons, and dendrites. **B.** Genes grouped into Biological Processes that are most upregulated in GUCY2C^{High} cells are related to hormone transport, peptide release, and cell-cell signaling. **C.** Genes grouped into molecular function that are most upregulated in GUCY2C^{High} cells are related to calcium ion binding (like synaptotagmins), hormones, neuropeptides, receptor activity, and ion channel activity. GO profiling analyzed using gprofiler in R Studio.

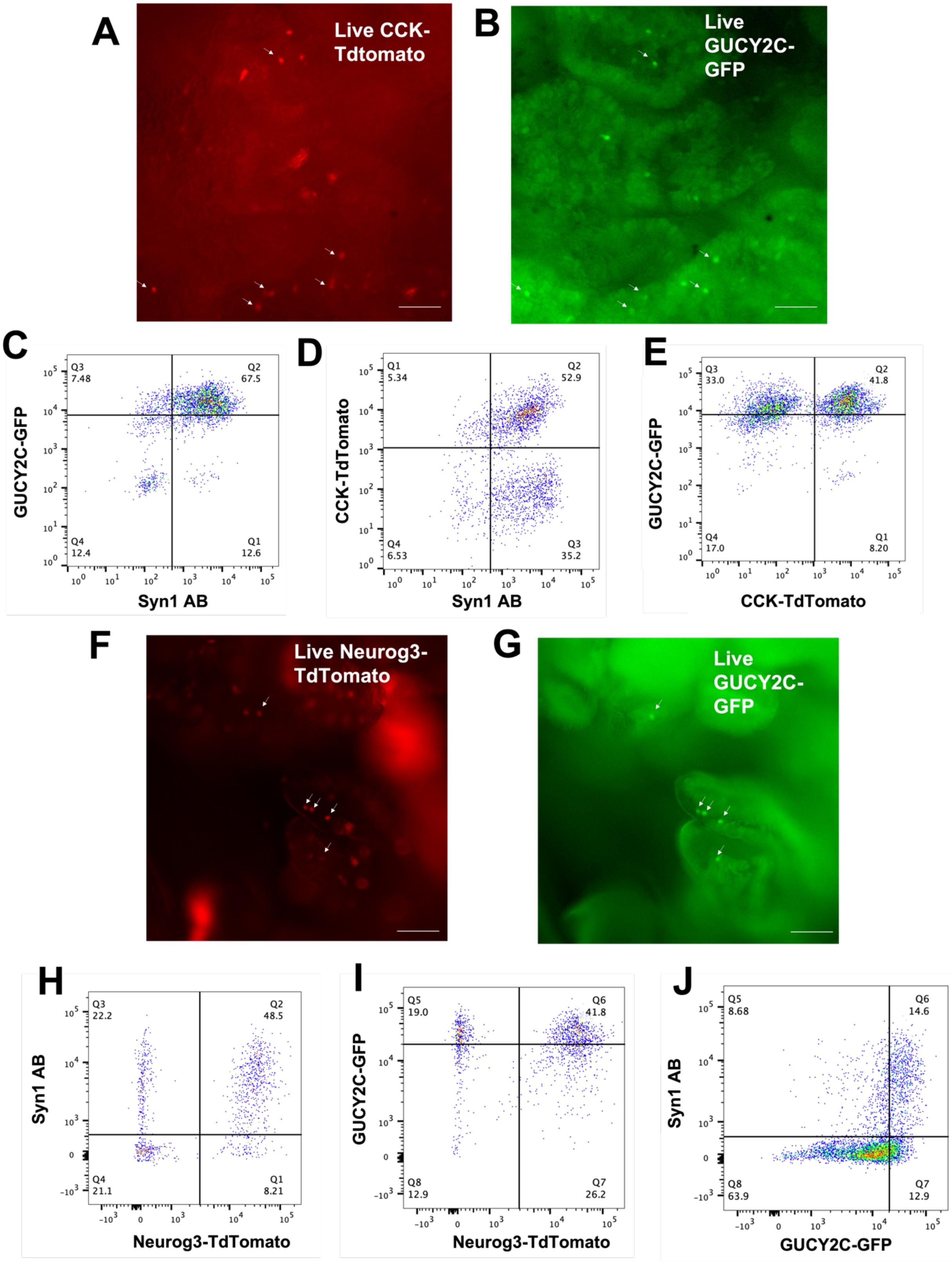

A
Live CCK-Tdtomato
B
Live GUCY2C-GFP
C
Q3 7.48
Q2 67.5
Q4 12.4
Q1 12.6
GUCY2C-GFP
Syn1 AB
D
Q1 5.34
Q2 52.9
Q4 6.53
Q3 35.2
CCK-TdTomato
Syn1 AB
E
Q3 33.0
Q2 41.8
Q4 17.0
Q1 8.20
GUCY2C-GFP
CCK-TdTomato
F
Live Neurog3-TdTomato
G
Live GUCY2C-GFP
H
Q3 22.2
Q2 48.5
Q4 21.1
Q1 8.21
Syn1 AB
Neurog3-TdTomato
I
Q5 19.0
Q6 41.8
Q8 12.9
Q7 26.2
GUCY2C-GFP
Neurog3-TdTomato
J
Q5 8.68
Q6 14.6
Q8 63.9
Q7 12.9
Syn1 AB
GUCY2C-GFP

Supplemental Figure 5. A majority of GUCY2C^{High} cells overlap with neuropod and enteroendocrine reporter mouse lines. A. *CCK-cre Tdtomato* mouse crossed with **B.** *GUCY2C-GFP* mouse shows overlap of Tdtomato and GFP+ intestinal cells. **C.** CCK-Tdtomato high cells are enriched for GUCY2C (GFP) and Syn1 (APC). **D.** GUCY2C-GFP high cells are enriched for Syn1 and CCK-Tdtomato. **E.** Syn1 high cells are enriched for GUCY2C and CCK-Tdtomato. **F.** *Ngn3-cre Tdtomato* mouse crossed with **G.** *GUCY2C-GFP* mouse shows overlap of Tdtomato and GFP+ intestinal cells. **H.** GUCY2C-GFP high cells are enriched in both Syn1 (APC) and developmentally express enteroendocrine marker Ngn3-Tdtomato (PE). **I.** Syn1 high cells are enriched in GUCY2C and Ngn3. **J.** A subset of Ngn3-Tdtomato enteroendocrine cells express GUCY2C and Syn1. Scale bar = 50 μm

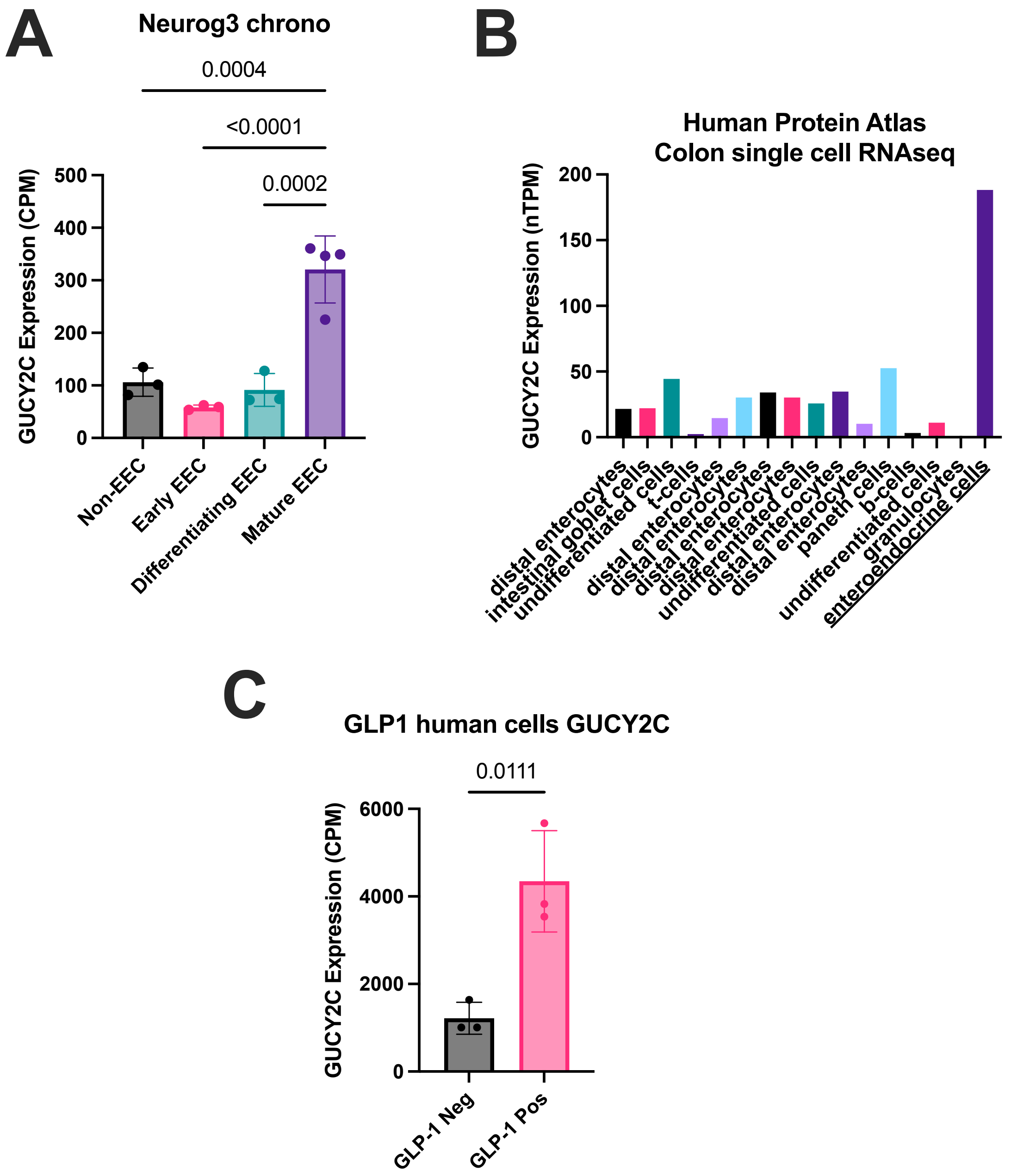

Supplemental Figure 6. GUCY2C is upregulated in mouse and human enteroendocrine cells. **A.** Bulk RNAseq from Neurog3 Chrono Mice reveals that GUCY2C is upregulated only in mature enteroendocrine (EEC) cells in the intestine (accession: GSE113561)(1). **B.** Single cell RNAseq of the human colon reveals that GUCY2C is upregulated in the enteroendocrine cluster of cells (https://www.proteinatlas.org/ENSG00000070019-GUCY2C/single+cell+type/colon)(2). **C.** Bulk RNAseq from human GLU-Venus (GLP-1) organoids reveals that GUCY2C is upregulated in GLP-1-positive human intestinal epithelial cells (accession:[dataset] GSE148224)(3).

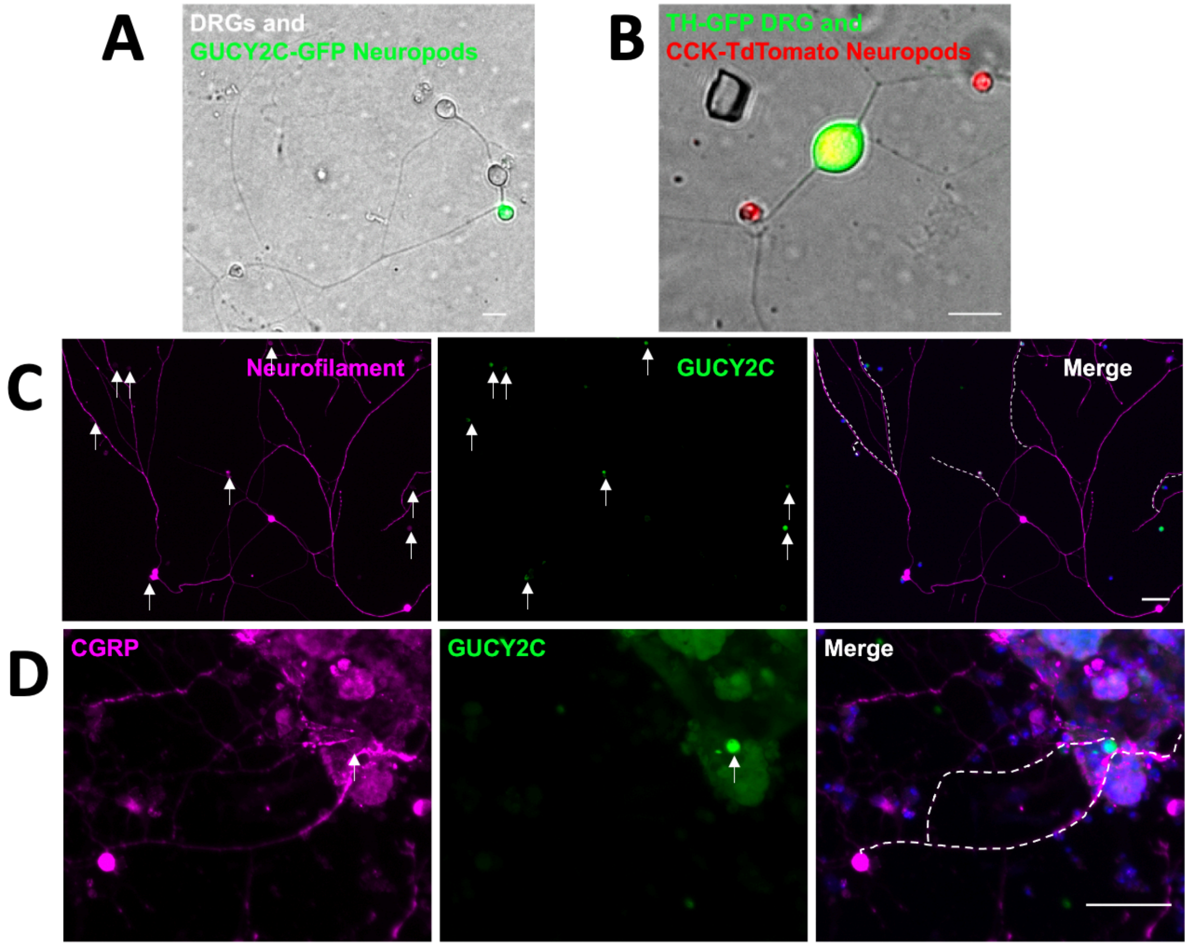

Supplemental Figure 7. GUCY2C^{High} Neuropod cells associate with DRG neurons in co-culture. A. Live imaging of Embryonic (E15.5) Dorsal Root Ganglia neurons (DRGs) and FACS sorted GUCY2C-GFP^{High} cells show that DRGs grow out to GUCY2C-GFP^{High} cells in culture. **B.** Similarly, E15.5. DRGs from *TH-GFP* mice grow out to CCK-TdTomato neuropod cells. **C.** Immunofluorescence of DRG neurons and sorted GUCY2C^{High} (arrows) co-cultures shows that Neurofilament+ DRG neurons grow out towards GUCY2C^{High} neuropod cells in culture (axons growing to GUCY2C^{High} cells highlighted with hashed white line). **D.** Immunofluorescence of DRG neurons and intestinal crypts show that CGRP+ DRG neurons grow out specifically towards GUCY2C^{High} neuropod cells (arrow) in intestinal crypts (axons growing to GUCY2C^{High} cells highlighted with hashed white line). (Scale bar = 50 μm)

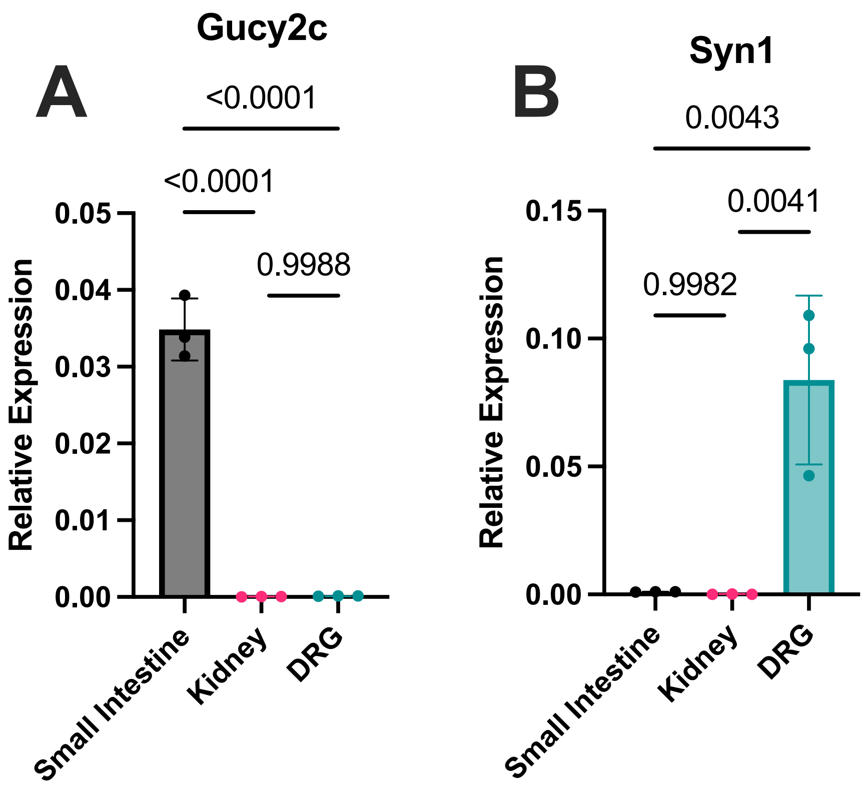

Supplemental Figure 8. Gucy2c is not expressed in DRG neurons. A. qRT-PCR for GUCY2C shows that embryonic (E15.5) Small intestine is positive for GUCY2C+ transcripts, whereas kidney and DRG neurons are not (n=3). **B.** qRT-PCR for neuronal marker Syn1 shows that E15.5 DRG neurons are significantly Syn1 positive, while E15.5 Small intestine and kidney are not (n=3).

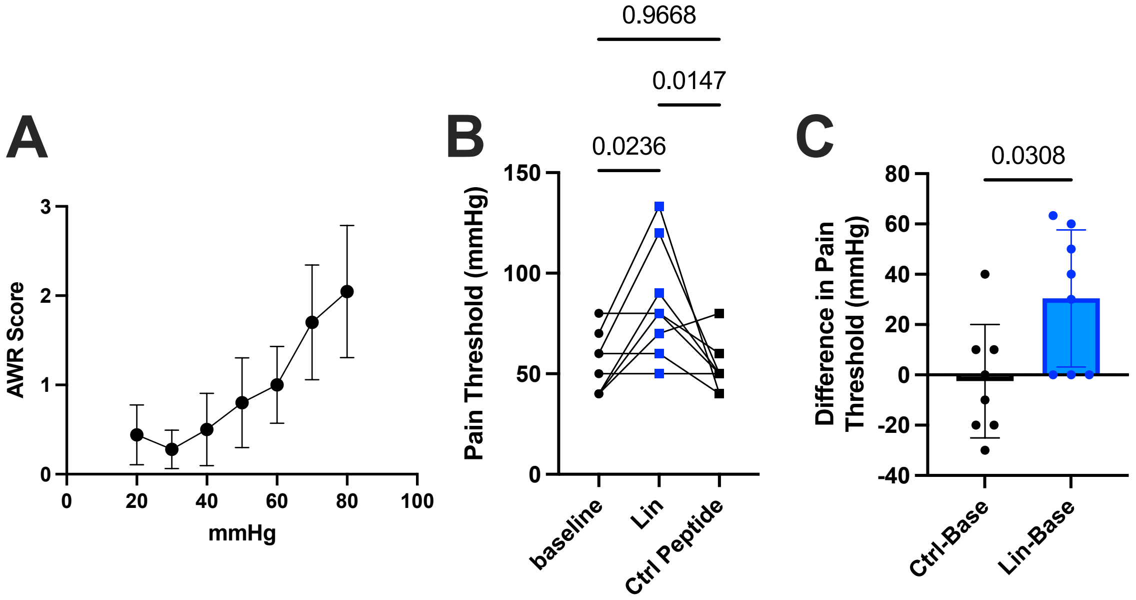

Supplemental Figure 9. Pain responses increase with increasing colorectal distention (CRD) and pain threshold is increased with linaclotide treatment. A. Increasing CRD pressure increases average pain responses in C57/Bl6J mice (n=5). **B.** Pain threshold (defined as an AWR score of 3 or above) is increased with linaclotide (Lin) administration and unchanged with control peptide (Ctrl; n=8). **C.** Difference in pain threshold after treatment with 2 mg/kg linaclotide or 2 mg/kg control peptide (n=8)

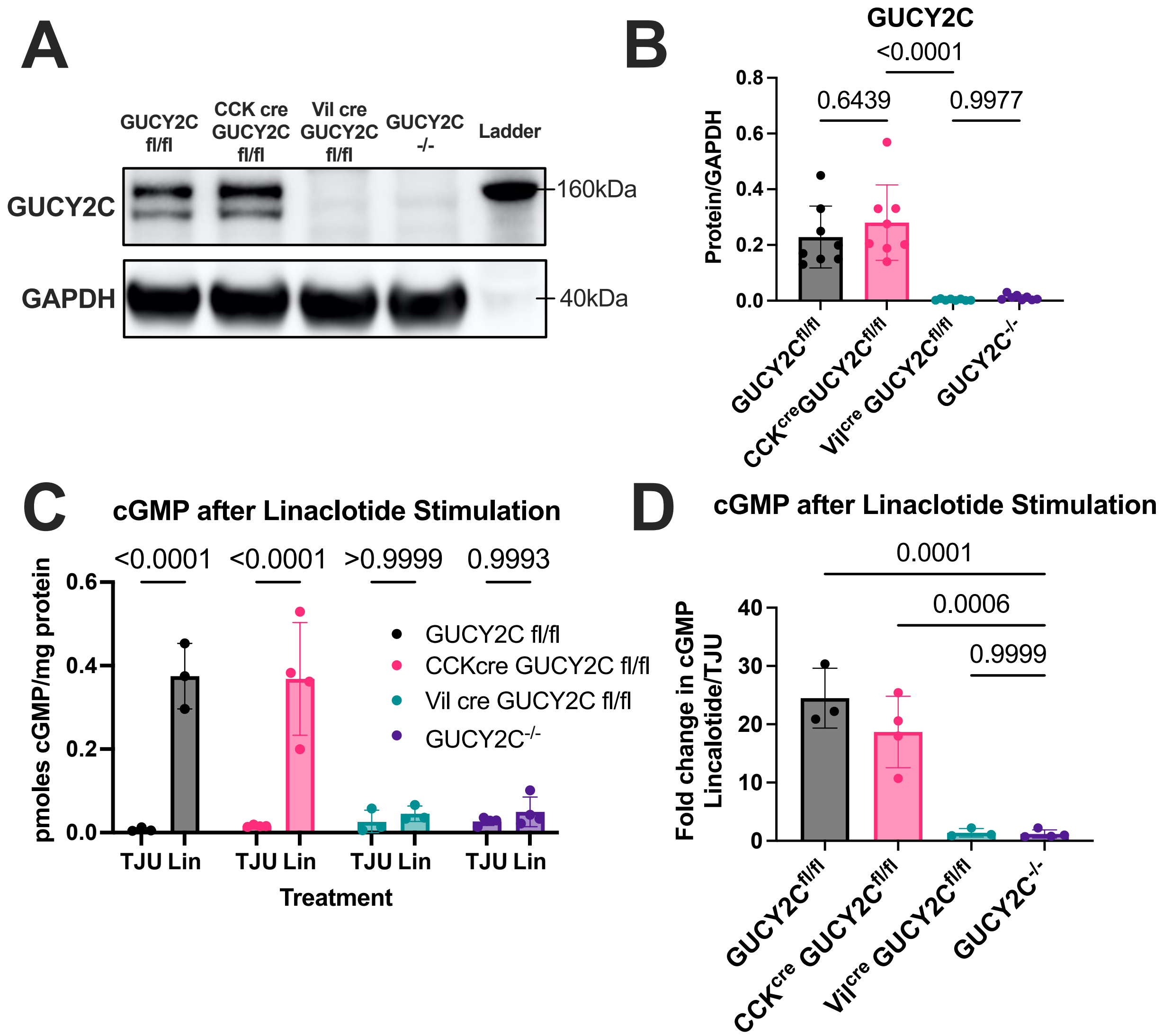

Supplemental Figure 10. Organoids from *GUCY2C$^{fl/fl}$* mice produce extracellular cGMP when stimulated with linaclotide. A. Immunoblot of small intestinal organoids produced from *GUCY2C$^{fl/fl}$* mice show that *CCK^{cre}GUCY2C$^{fl/fl}$* maintain GUCY2C expression (because CCK+ cells are <1% of intestinal cells), but *Vil^{cre}GUCY2C$^{fl/fl}$* mice lose GUCY2C expression similar to *GUCY2C$^{-/-}$* mice (because 100% of small intestinal cells are Vil+). **B.** Quantification of immunoblots from organoids grown from mice of each respective genotype (n=8). **C.** ELISA of extracellular cGMP release reveals that organoids derived from *GUCY2C$^{fl/fl}$* and *CCK^{cre}GUCY2C$^{fl/fl}$* react to 1 μM linaclotide (24 h stimulation) by producing extracellular cGMP, while organoids derived from *Vil^{cre}GUCY2C$^{fl/fl}$* and *GUCY2C$^{-/-}$* mice do not produce cGMP (n=3-4) **D.** Data in C normalized to the inactive peptide TJU, showing that *GUCY2C$^{fl/fl}$* organoids react to linaclotide with cGMP production. (n=3-4 mice, statistics from one-way ANOVA with multiple comparisons).

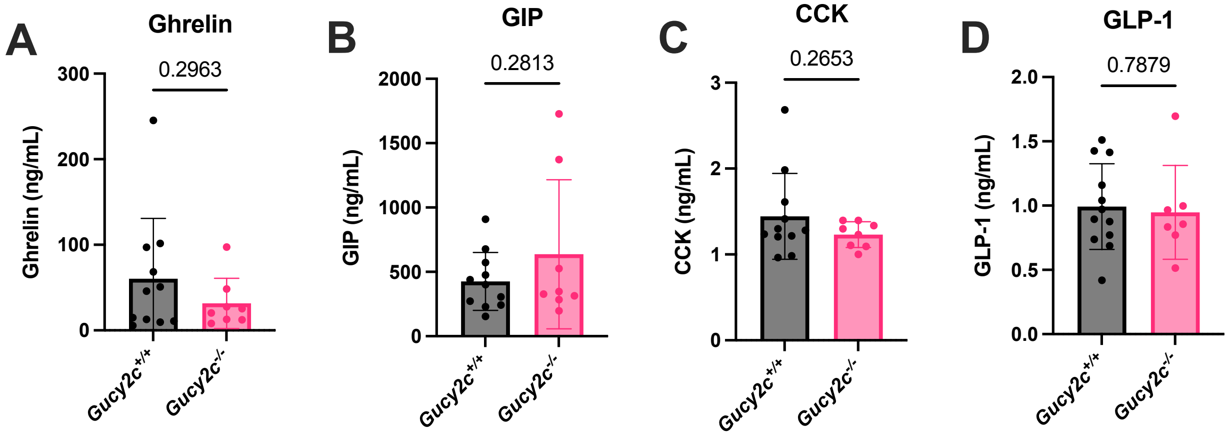

Supplemental Figure 11. Enteroendocrine hormones are not significantly different between Gucy2c$^{-/-}$ and Gucy2c$^{+/+}$ mice. A and B. Ghrelin and GIP were measured in mouse serum by Eve technologies Mouse Metabolic Multiplex assay, and were not significantly different in the two genotypes in blood 2 h after refeeding following a 16 h fast. **C and D**. CCK and GLP-1 were measured in mouse serum by ELISA (Phoenix Pharmaceuticals) 2 h after refeeding following a 16 h fast. These hormones, as well, were not different between the genotypes, indicating that absence of GUCY2C does not change endocrine hormone function of enteroendocrine cells. (n=7-11 mice/group, statistics from 2-sided unpaired students t-test)

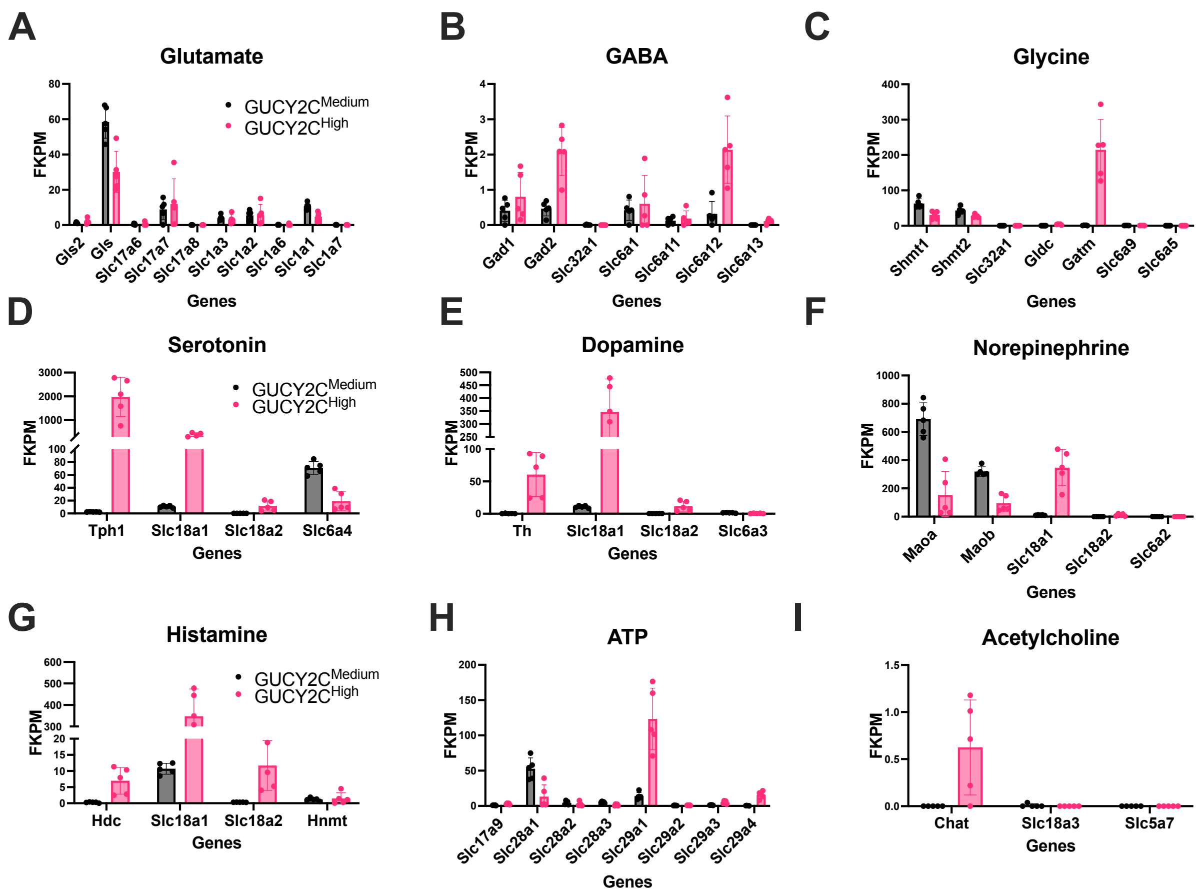

Supplemental Figure 12. Analysis of neurotransmitter gene expression reveals that GUCY2C^{High} cells most highly express monoamine machinery. A. Transcripts for glutamate synthesis, packaging, and reuptake. **B.** Transcripts for GABA synthesis, packaging, and reuptake. **C.** Transcripts for glycine synthesis, packaging, degradation, and reuptake. **D.** Transcripts for serotonin (monoamine) synthesis, packaging, and reuptake. **E.** Transcripts for dopamine (monoamine) synthesis, packaging, and reuptake. **F.** Transcripts for norepinephrine (monoamine) synthesis, packaging, and reuptake. **G.** Transcripts for histamine synthesis, packaging, and degradation. **H.** Transcripts for ATP packaging and reuptake. **I.** Transcripts for acetylcholine synthesis, packaging, and reuptake.

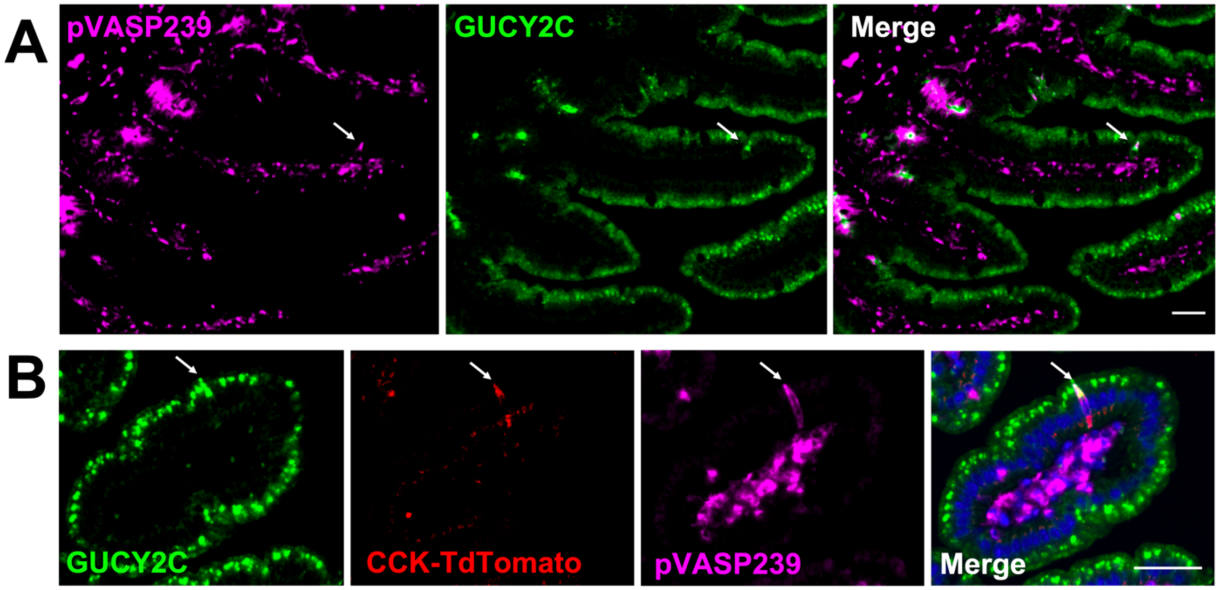

Supplemental Figure 13. GUCY2C^{High}CCK^{High} cells are enriched in pVASP239, a downstream marker of GUCY2C activity. A. pVASP239 staining overlaps with GUCY2C in the intestinal crypt and specifically in GUCY2C^{High} cells in the intestinal villus. **B.** pVASP239 staining overlaps with CCK-TdTomato cells that are also GUCY2C^{High}. Images taken from jejunum. (Scale bar = 50 μm)

Supplemental Methods

Small intestinal crypt preparation and organoid culture

Organoids were prepared as described (4, 5). Briefly, mice underwent cervical dislocation, intestines dissected and placed in ice-cold PBS, flushed with a 20G needle and 20 mL syringe, and opened longitudinally. Villi were scraped off using a glass coverslip and intestines were placed whole into 10 mL dissociation buffer (DMEM without Ca^{2+} and Mg^{2+} (Corning, 10-013-CV), 10 mM EDTA (Invitrogen, AM9261), 10% fetal bovine serum (Cytiva, SH30071.03)). Intestines were rotated in a MACS rotator at 4°C for 1 h, after which intestines were shaken and vortexed for 3 min to dislodge epithelial cells. Intact intestine was discarded and epithelium in the supernatant was spun down at 500 rcf for 5 min at 4°C, then filtered through a 100 µm filter. Crypts were then separated by sequential differential centrifugation: first in 10% w/v sorbitol (Sigma Aldrich, St. Louis, MO, S1876) at 500 rcf, then 3% percoll (Cytiva, 17089102) at 1500 rcf, and finally in 10% sorbitol at 500 rcf. Isolated crypts were washed by centrifugation in DMEM, then counted for plating in Matrigel. 200 crypts per well were plated in 50 µL of Growth Factor Reduced Matrigel (Corning, 356234). Matrigel was allowed to solidify at 37°C for 20 min, then 500 µL of 37°C IntestiCult OGM mouse basal medium (StemCell Technologies, 06005) supplemented with 10 µM Y-27632 rock inhibitor (StemCell Technologies, 72302), and 1 µM neural growth factor (NGF) (Peprotech, 450-34) was added to each well. Organoids were maintained at 37°C with 5% CO2, media was replaced every 3 days, and organoids were split every 7 days with TrypLE (Gibco, 12605010). Experimental organoids were used 3 days after the first split.

Cyclic GMP ELISA

Cyclic GMP was detected on sorted cells using a Cyclic GMP ELISA Kit (Cayman Chemical, 581021). The assay was performed according to manufacturer's instructions with the following modifications: acetylated samples were diluted 1-fold in cGMP ELISA Buffer and the standard curve was performed with 3-fold serial dilutions instead of 2-fold to achieve the lowest detectable concentration of cGMP.

Cyclic GMP production was induced in cells after sorting into 100 μL of Neurobasal+ media in 1 mM IBMX (Sigma Aldrich, I5879) to inhibit phosphodiesterase activity. Cells were treated with 1 μM linaclotide (Ironwood Pharmaceuticals), or 1 μM inactive peptide TJU (inactive analog ST(5–17)Ala,9,17Cys(Acm),5,10 6-14 disulfide; Bachem, Bubendorf, Switzerland) for 1 h before spinning down cells and flash freezing supernatants and cell pellets.

Organoids for cGMP analysis also were treated with 1 mM IBMX for 20 min prior to treatment with 1 μM linaclotide or TJU for 24 h. After incubation, supernatants were assessed for cGMP, while organoids were isolated from Matrigel in Cell Recovery Solution (Corning, 354253). Cyclic GMP production was normalized to protein content of organoids in each well. At least 4 wells/ condition were used in organoid experiments, and each point on the graph represents the average of wells for one mouse of that genotype.

RNAseq Library Preparation

Briefly, mRNA was purified from total RNA after QC using poly-T oligo-attached magnetic beads. Fragmentation was carried out using divalent cations under elevated temperature in NEBNext First Strand Synthesis Reaction Buffer (5X) (NEB, E7525). First strand cDNA was synthesized using random hexamer primer and M-MuLV Reverse Transcriptase (RNase H-) (NEB, M0253). Second

strand cDNA synthesis was subsequently performed using DNA Polymerase I and RNase H. Remaining overhangs were converted into blunt ends via exonuclease/polymerase activities. After adenylation of 3' ends of DNA fragments, NEBNext Adaptor (NEB, E6442) with hairpin loop structure was ligated to prepare for hybridization. To select cDNA fragments of preferentially 150~200 bp in length, the library fragments were purified with AMPure XP system (Beckman Coulter). Then 3 µl USER Enzyme (NEB, M5505) was used with size-selected, adaptor ligated cDNA at 37°C for 15 min followed by 5 min at 95°C before PCR. Then PCR was performed with Phusion High-Fidelity DNA polymerase (NEB, M0530), Universal PCR primers and Index (X) Primer (NEB, E7335). Finally, PCR products were purified (AMPure XP system) and library quality was assessed on the Agilent Bioanalyzer 2100 system. The clustering of the index-coded samples was performed on an Illumina Novaseq6000 PE150 sequencer according to the manufacturer's instructions. After cluster generation, the libraries were sequenced on the same machine and paired-end reads were generated. STAR software was then used to align and map clean reads to the mouse reference genome (mm9).

Measurement of Intestinal Hormones

Intestinal hormones were collected from *Gucy2c*$^{-/-}$ and *Gucy2c*$^{+/+}$mice 2h after refeeding following an overnight (16h) fast. Blood was collected via eye bleeds using non-heparinized glass capillary tubes collecting 200-300uL of blood per mouse. Proteinase inhibitors were added to prevent degradation of target proteins: Pefabloc SC (Roche, 11429868001), DPP IV Inhibitor (Millipore, DPP4-010), and Protease Inhibitor Cocktail (Sigma, P2714). Samples were placed on ice immediately after collection and spun down at 4000 g (at 4°C) to obtain serum without RBCs, and stored at -80°C prior to assay. Ghrelin and GIP were measured using the Mouse, Rat Metabolic Array by Eve Technologies (Calgary, AB, Canada). CCK and GLP-1 were measured using: CCK

EIA (26-33) Immunoassay Kit (Phoenix Pharmaceuticals, EK-069-04), and GLP-1 EIA (7-36) Immunoassay Kit (Phoenix Pharmaceuticals, EK-028-11). All serum was assayed according to company/kit instructions.

Immunoblot

Immunoblot of mouse small intestinal organoids was performed by dissolving Matrigel, then extracting protein from organoids. Matrigel was dissolved using Cell Recovery Solution (Corning, 354253) at 500 uL/well according to manufacturer's instructions. Organoids liberated from Matrigel were extracted in T-Per (Thermo Fisher Scientific, 78510) supplemented with protease and phosphatase inhibitors (Roche, 04693116001 & 4906837001). Twenty micrograms of protein were loaded into each lane, and membranes were cut so that the same membrane was used to quantify GAPDH (1:5000) and GUCY2C (1:1000) for each sample. Membranes were incubated with HRP-conjugated secondary antibodies (1:25000) and developed using Supersignal West Dura Substrate (Thermo Fisher, 34075). Images were captured on the BioRad ChemiDoc MP imaging station, and bands were quantified using densitometry normalized to GAPDH on ImageJ software.

Avertin preparation

Avertin was prepared as a stock solution of 10 g in 10 mL of 2-methyl-2-butanol (Sigma Aldrich, 152463), diluted to a 25 mg/mL working solution in PBS, and injected IP at 1 μg/kg.

Supplemental Table 1. Antibodies

Antibody	Target	Source	Identifier	Species/ Isotype	Concentration
**Primary Antibodies**	Phospho p44/42 MAPK (Erk1/2)	Cell Signaling	4370	Rabbit	1:200
	GUCY2C (MS20)	In House(6)	N/A	Mouse IgG2a	1:1000
	PrEST GUCY2C	Millipore Sigma	APREST79587	Rabbit	1:1000
	GLP-1 (7-36) Antibody	Phoenix Pharmaceuticals	H-028-13	Rabbit	1:1000
	Synapsin 1 (D12G5)	Cell Signaling	5297	Rabbit	1:300
	phospho VASP (Ser239)	Millipore Sigma	SAB4300129	Rabbit	1:1000
	GFP	Abcam	13970	Chicken	1:500
	Synaptophysin (YE269)	Abcam	32127	Rabbit	1:1000
	Beta III Tubulin	Abcam	18207	Rabbit	1:1000
	GUCY2C	Sigma-Aldrich	HPA037655	Rabbit	1:1000
	CD326 (Epcam), APC conjugated	Miltenyi Biotech	130-123-810	Rat IgG1	1:1000
	CGRP	Millipore Sigma	PC205L	Rabbit	1:1000
	Neurofilament	Abcam	ab4680	Rabbit	1:500
	Pgp9.5	Abcam	ab108986	Rabbit	1:500
	Beta Catenin	Santa Cruz	sc-7963	Mouse IgG1	1:500
	Rabbit IgG Isotype Control	Invitrogen	02-610-2	Rabbit	1:2000
**Secondary Antibodies**	DAPI	Thermo Fisher	D1306		1:4000
	Donkey Anti-Rabbit IgG; Alexa Fluor 488	Jackson ImmunoResearch	711-545-152		1:200

	Donkey Anti-Chicken IgY; Alexa Fluor 594	Jackson ImmunoResearch	703-585-155		1:500
	Goat Anti-Rabbit IgG; Alexa Fuor Plus 647	Thermo Fisher	A-32733		1:1000
	Goat Anti-Mouse IgG, Fcy subclass 1 specific, Alexa Fluor 594	Jackson ImmunoResearch	115-585-205		1:500
	Peroxidase Goat Anti-Mouse IgG, Fcy subclass 2a specific	Jackson ImmunoResearch	115-035-206		1:1000

Supplemental Table 2. Taqman Primer Probes

Gene	Taqman Probe
**Gucy2c**	Mm01267705_m1
**Syn1**	Mm00449772_m1
**Cck**	Mm00446170_m1
**Pyy**	Mm00520716_g1
**Dlg4.**	Mm00492193_m1
**Gapdh**	Mm99999915_g1

Supplemental References:

1. Gehart H, van Es JH, Hamer K, Beumer J, Kretzschmar K, Dekkers JF, et al. Identification of enteroendocrine regulators by real-time single-cell differentiation mapping. *Cell.* 2019;176(5):1158-73. e16.
2. Karlsson M, Zhang C, Méar L, Zhong W, Digre A, Katona B, et al. A single–cell type transcriptomics map of human tissues. *Science Advances.* 2021;7(31):eabh2169.
3. Goldspink DA, Lu VB, Miedzybrodzka EL, Smith CA, Foreman RE, Billing LJ, et al. Labeling and characterization of human GLP-1-secreting L-cells in primary ileal organoid culture. *Cell reports.* 2020;31(13):107833.
4. Pattison AM, Blomain ES, Merlino DJ, Wang F, Crissey MAS, Kraft CL, et al. Intestinal enteroids model guanylate cyclase C-dependent secretion induced by heat-stable enterotoxins. *Infection and immunity.* 2016;84(10):3083-91.

5. Sato T, Vries RG, Snippert HJ, Van De Wetering M, Barker N, Stange DE, et al. Single Lgr5 stem cells build crypt-villus structures in vitro without a mesenchymal niche. *Nature.* 2009;459(7244):262-5.
6. **Merlino D, Barton J**, Charsar B, Byrne M, Rappaport J, Smeyne R, et al. Two distinct GUCY2C circuits with PMV (hypothalamic) and SN/VTA (midbrain) origin. *Brain Structure and Function.* 2019;224(8):2983-99.

Author names in bold designate shared co-authorship
